# Association of sleep quality and sleep duration with anxiety symptoms in older adults: a systematic review and meta-analysis

**DOI:** 10.3389/fpsyt.2026.1838523

**Published:** 2026-05-28

**Authors:** Junjie Zhang, Xiaochun Zhu, Lei Zhang, Yanmin Shen, Meili Shi, Haiying Min

**Affiliations:** Clinical Research Center for Mental Disorders, Shanghai Pudong New Area Mental Health Center, School of Medicine, Tongji University, Shanghai, China

**Keywords:** anxiety, meta-analysis, older adults, sleep duration, sleep quality, systematic review

## Abstract

**Background:**

Sleep disturbances are highly prevalent among older adults, with prevalence ranging from 40–70%. While numerous meta-analyses have examined the association between sleep and depression, the relationship between sleep problems and anxiety—particularly in older adults—has received comparatively less attention. Direct meta-analytic comparisons between sleep quality and sleep duration in relation to anxiety among older adults remain limited.

**Methods:**

We searched PubMed, Web of Science, Embase, Cochrane Library, and CNKI from inception to January 2026 according to the PRISMA 2020 guidelines. The studies that provided the odds ratios (ORs) with 95% confidence intervals (CIs) for the associations between sleep quality/disturbance or sleep duration and anxiety in adults aged 60 years or older were included. The DerSimonian–Laird random-effects model was used to synthesize the pooled ORs. Subgroup analysis, meta-regression, sensitivity analyses, and publication bias assessment were performed.

**Results:**

A total of 19 studies with 70,716 participants were included in this meta-analysis and 21 effect-size records were available. Poor sleep quality (OR = 4.00, 95% CI: 2.96–5.41; *I*^2^ =93.4%; *k* = 16) and short sleep duration (OR = 2.14, 95% CI: 1.85–2.46; *I*^2^ = 39.4%; *k* = 5) were significantly associated with anxiety among older adults. Compared with short sleep duration, poor sleep quality showed a larger pooled association (*z* = 3.69, *P <* 0.001; OR ratio = 1.87). Associations remained significant across subgroups defined by geographic region, study design, and anxiety measurement tool. The leave-one-out ORs ranged from 3.68 to 4.26. The trim-and-fill analysis indicated that only one missing study was required to balance the funnel plot. Substantial heterogeneity in the sleep quality analysis (*I*^2^ = 93.4%) reflected differences in measurement instruments and geographic regions.

**Conclusion:**

Poor sleep quality and short sleep duration were both associated with anxiety in older adults, with a larger but more heterogeneous association for sleep quality. These findings support routine assessment of sleep quality in older adults with anxiety symptoms. Prospective studies are needed to clarify directionality.

**Systematic review registration:**

https://www.crd.york.ac.uk/prospero/, identifier CRD420261348264.

## 1 Introduction

The global population is ageing rapidly, and the number of people aged 60 years or over is projected to double by 2050 (1). Sleep problems are highly prevalent in this demographic, affecting an estimated 40–70% of older adults (2, 3). Several age-related changes contribute to this burden, including a reduction in slow-wave sleep, increased nocturnal wakefulness, and an advance in the circadian clock. These changes can affect perceived sleep quality even when total sleep time is not markedly reduced. Sleep quality refers to the subjective and multidimensional sleep experience, including satisfaction, continuity, efficiency, and restorative value, and is commonly assessed using instruments such as the Pittsburgh Sleep Quality Index (PSQI). Sleep duration refers to total sleep time per night. These constructs are related but clinically distinct: sleep quality captures perceived continuity and restorative sleep, whereas sleep duration captures quantity. Despite the clinical relevance of both dimensions, research examining them in parallel remains limited.

Anxiety is another important yet frequently overlooked aspect of geriatric mental health. The prevalence of anxiety disorders among community-dwelling older adults has been estimated at 3.2–14.2%, with rates varying considerably across diagnostic criteria and study populations (4). Anxiety in late life is often under-detected, partly because somatic symptoms may be attributed to coexisting physical illness and psychological distress may go unacknowledged. Untreated late-life anxiety is associated with a range of adverse outcomes, including accelerated cognitive decline, cardiovascular morbidity, functional impairment, and suicidal ideation (4). Identifying modifiable risk factors is therefore essential for developing targeted interventions that improve the well-being of older adults experiencing anxiety-related symptoms.

A growing body of epidemiological evidence links sleep problems to anxiety across the lifespan. Experimental and neuroimaging research suggests that sleep loss amplifies amygdala reactivity and disrupts prefrontal regulatory control, producing a state of heightened emotional vulnerability (5). Several meta-analyses have examined the broader relationship between sleep and mental health. Baglioni et al. (6) reported a strong association between insomnia and incident depression (OR = 2.60) based on longitudinal studies, and Bao et al. (7) reported that persistent sleep disturbances predicted incident depression in older adults (pooled RR = 1.92). Despite this progress, several critical gaps remain. First, no existing meta-analysis examines the sleep–anxiety relationship exclusively within older adult populations; current syntheses either include younger age groups or focus on the sleep– depression relationship. Second, whereas sleep quality has been the primary exposure variable in prior work, sleep duration has received comparatively little attention. Third, no quantitative synthesis has directly compared the strength of association between sleep quality and sleep duration as predictors of anxiety. Finally, considerable between-study heterogeneity in the operationalisation of sleep disturbance—including differences in measurement instruments, cut-off values, and diagnostic criteria—has yet to be systematically explored as a source of variation in the context of anxiety in older adults.

This study was designed to address these gaps by conducting a systematic review and meta-analysis of the associations between sleep quality, sleep duration, and anxiety symptoms in adults aged 60 years and older. The study makes three principal contributions. First, it provides age-specific meta-analytic evidence on the sleep–anxiety relationship in older populations, yielding estimates that can be compared with those from general-population syntheses. Second, by examining sleep quality and sleep duration as parallel exposures, it enables a direct comparison of their respective effect sizes and informs clinical prioritisation. Third, it employs a comprehensive analytical framework, including subgroup analyses across five dimensions, meta-regression, and multiple sensitivity and publication bias assessments, thereby exploring how differences in exposure and outcome measurement contribute to between-study variation.

## 2 Methods

### 2.1 Protocol and registration

This systematic review and meta-analysis adhered to the PRISMA 2020 guidelines (8) and the MOOSE reporting checklist (9). The protocol of this study was registered in the International Prospective Register of Systematic Reviews (PROSPERO; CRD420261348264). Please see **Supplementary Table 2** for the PRISMA 2020 checklist.

### 2.2 Search strategy

We searched PubMed, Web of Science, Embase, Cochrane Library and CNKI from inception through January 2026. No language restrictions were applied at the initial search stage.

Boolean operators were used among the three term groups. The first group was related to sleep exposures and included “sleep quality”, “sleep duration”, “sleep disturbance”, “insomnia”, “Pittsburgh Sleep Quality Index” and “PSQI”. The second group was related to anxiety and included “anxiety”, “anxious”, “generalized anxiety disorder”, “GAD” and “anxiety symptoms”. The third group was related to the target population and included “elderly”, “older adults”, “aged”, “geriatric”, “senior” and age-related terms (such as “≥60 years”). MeSH terms and Emtree terms were matched with free-text words in PubMed and Embase to maximize sensitivity, and the Chinese subject terms were used in CNKI.

In addition to electronic searching, we screened the reference lists of included studies and review articles to identify any further publications that may have met our criteria. For details of the search strategy for each database, including the search terms used and the number of records identified, see **Supplementary Table 4** (10).

### 2.3 Eligibility criteria

The PECO (Population, Exposure, Comparator, and Outcome) framework was used to identify relevant studies.

Population: Studies conducted with community-dwelling or institutionalized older adults. This means that the studies included participants on average 60 years or older, or that the study authors described them as “the elderly”.

Exposure: Poor sleep quality or sleep disturbance was defined using the operational categories reported in the original studies, including multidimensional sleep quality scales such as the Pittsburgh Sleep Quality Index (PSQI), insomnia-specific scales such as the Insomnia Severity Index (ISI) and Athens Insomnia Scale (AIS), clinical or self-reported insomnia, and self-reported sleep problems. This grouping reflects the overlap among poor sleep quality, sleep disturbance, and insomnia symptoms in older adults, while recognizing that these measures are not identical constructs (11). Short sleep duration was defined according to the thresholds used in the original studies, mainly <6, ≤6, or <7 hours per night.

Comparator: A good sleep quality, absence of sleep disturbance or a reference category of sleep duration (typically 7–8 hours per night).

Outcome: Anxiety symptoms as measured by psychometric assessment tools such as Generalized Anxiety Disorder scale (GAD-7 or GAD-2), Self-Rating Anxiety Scale (SAS), Hospital Anxiety and Depression Scale–Anxiety subscale (HADS-A), Hamilton Anxiety Rating Scale (HAMA) or other validated anxiety assessment tools.

Studies were selected based on the following criteria: (1) study design—only observational studies including cross-sectional, cohort and case-control studies were allowed; (2) availability of ORs with corresponding 95% CI or at least enough information to derive them; and (3) English or Chinese language of publication.

Exclusion criteria: Studies involving interventions such as randomized controlled trials; studies on specific populations with diagnosed diseases (e.g. Parkinson’s disease, cancer, post-surgery); reviews, editorials, conference abstracts, case reports and letters; and studies that did not provide extractable data to estimate the OR. Depression was not used as an exclusion criterion, because the review targeted the sleep– anxiety association in older adult populations and most source studies did not provide extractable estimates stratified by depression status. Adjustment for depressive symptoms was extracted where available.

### 2.4 Study selection and data extraction

**Figure 1:**
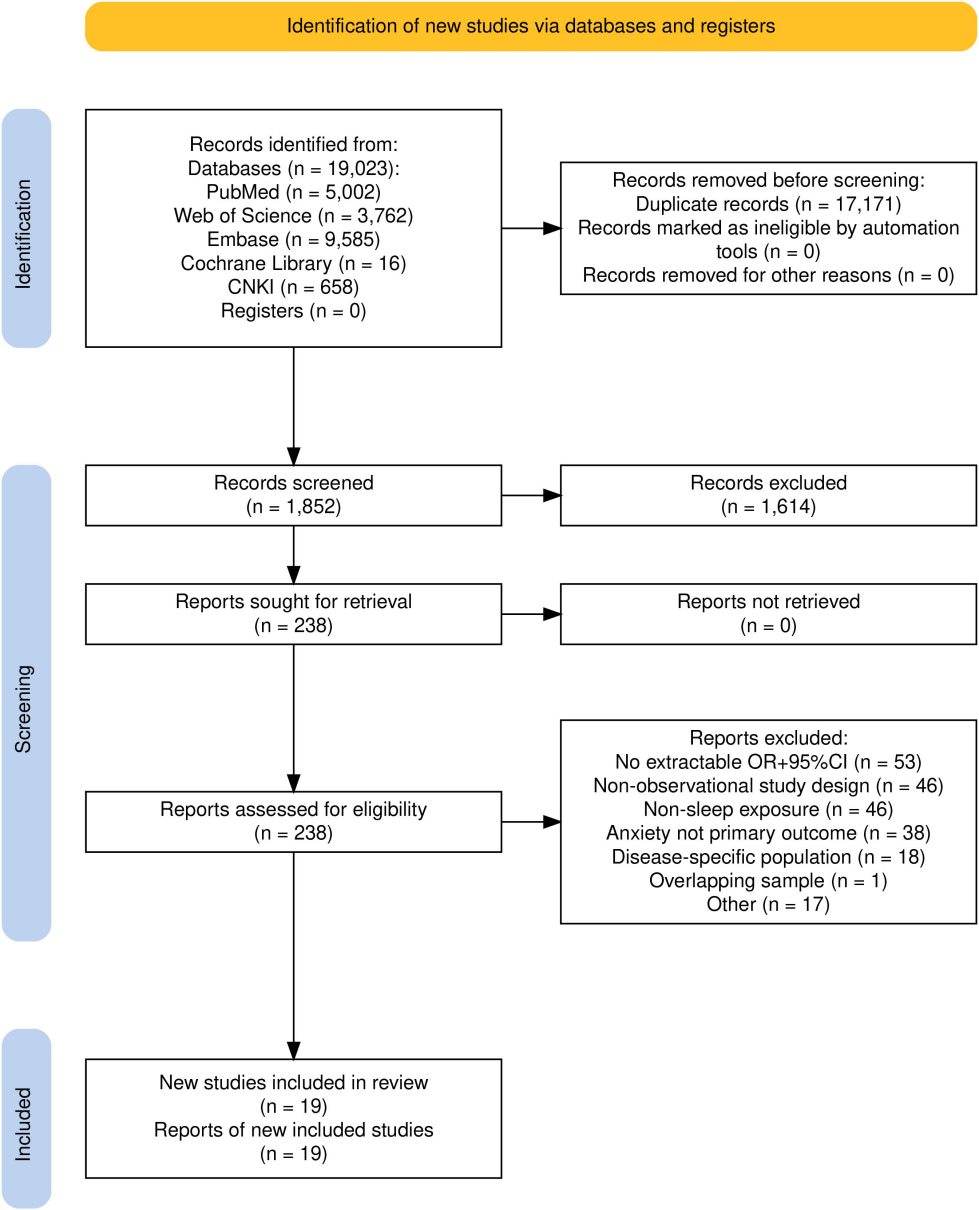
Study selection flow diagram according to the PRISMA 2020 statement. Five databases were interrogated to find relevant reports, resulting in 19,023 records. After initial screening of these for relevance, 19 full-text studies were selected for inclusion in the meta-analysis presented in this systematic review.

We performed two stages of study selection. Stage 1 consisted of two reviewers independently screening titles and abstracts. Stage 2 consisted of two reviewers independently assessing full text papers against our criteria. In the event of disagreement, studies were discussed to reach consensus, and if required, a third reviewer was consulted. The PRISMA 2020 flow diagram (**Figure 1**) is shown.

Two reviewers used a standardized data extraction form to assess, separately, the following study characteristics: first author, publication year, country, study design, number of participants, exposure criteria and measurement tool, cutoff value for exposure, anxiety assessment scale and threshold value, adjusted OR and 95% CI, and covariates adjusted for in multivariable analyses. For studies reporting ORs for both sleep quality and sleep duration (i.e., dual-exposure studies), effect sizes were extracted separately and entered into the corresponding exposure category. In the event of disagreement, the reviewers reached consensus.

### 2.5 Quality assessment

The methodological quality of the cross-sectional and cohort studies was evaluated using the Agency for Healthcare Research and Quality (AHRQ) cross-sectional study quality assessment tool for the crosssectional studies, and the Newcastle-Ottawa Scale (NOS) for the cohort studies. The AHRQ cross-sectional study tool consists of 11 items with a maximum score of 11 (12). The studies with a score of ≥8 were considered to be of high quality, the studies with a score of 6–7 were considered moderate quality, and the studies with a score of <6 were considered low quality. The NOS also has a maximum score of 9 (13). Studies with a score of ≥7 were considered to be of high quality, while studies with a score ranging from 4 to 6 were considered moderate quality.

Each study was reviewed by 2 reviewers and interrater reliability was assessed using a weighted kappa statistic. Please refer to **Supplementary Table 1** for further quality assessment details.

### 2.6 Statistical analysis

#### 2.6.1 Effect size synthesis

Pooled Odds Ratios (ORs) with 95% Confidence Intervals (CIs) were calculated using the DerSimonian– Laird (DL) random-effects model as the primary synthesis model to account for within-study and betweenstudy heterogeneity (14). Fixed-effect estimates were presented only as modelcomparison sensitivity analyses. These analyses were performed separately for sleep quality/disturbance and sleep duration.

#### 2.6.2 Heterogeneity assessment

Heterogeneity was assessed using Cochran’s *Q* test (*P <* 0.10 indicating significant heterogeneity) and *I*^2^, where values of 25%, 50% and 75% correspond to low, moderate and high heterogeneity, respectively (15). The 95% prediction interval (95% PI) was also calculated to estimate the expected range of true effects across different study settings (16).

#### 2.6.3 Subgroup analysis and meta-regression

This subgroup analysis was performed in the sleep quality subgroup (*k* = 16) according to the following five factors: country (China vs. non-China), study design (cross-sectional vs. cohort), study quality (high quality, ≥8 vs. moderate quality, score 6–7), anxiety scale (GAD-7/GAD-2 vs. SAS vs. other) and exposure measurement tool (PSQI vs. insomnia scales vs. single-item). The Z test for between-group differences was used, and the results were compared using *Q*_between_, and the threshold for statistical significance was set as *P <* 0.05.

We performed a random-effects meta-regression to investigate the sources of heterogeneity, using quality score, log-transformed sample size, and publication year as covariates. For each covariate, the coefficient (*β*), model heterogeneity statistic (*Q*_model_), *P* value, and proportion of variance explained (*R*^2^) were reported.

#### 2.6.4 Sensitivity analysis

To assess the stability of the pooled estimate, we performed eight sensitivity analyses: leave-one-out analysis (1), omitting one study at a time; omitting the studies with the highest and lowest odds ratios (ORs) (2); fixed-effect model comparison (3); restriction to high-quality studies (quality score ≥8) (4); excluding potentially overlapped estimates from the Chinese Longitudinal Healthy Longevity Survey (CLHLS) while retaining one CLHLS-derived study at a time (5); replacing the incidence-based OR with the prevalence-based OR in the cohort study (6); applying the Hartung–Knapp–Sidik–Jonkman (HKSJ) adjustment (17) for the confidence interval of the pooled estimate (7); and cumulative meta-analysis ordered by the publication year (8).

#### 2.6.5 Publication bias

We assessed publication bias graphically with a funnel plot and statistically with Egger’s linear regression test (18) and Begg’s rank correlation test (19). If the trim-and-fill method (20) indicated that the asymmetry was due to unpublished studies rather than other causes, we recorded the number of studies estimated to be missing by the method, and recalculated the pooled OR with the missing studies included.

#### 2.6.6 Evidence quality

We graded the overall certainty of the evidence using the GRADE approach, addressing potential risk of bias, heterogeneity, indirectness, imprecision and publication bias (21). The GRADE evidence profile is shown in **Supplementary Table 3**.

#### 2.6.7 Software

All statistical analyses were performed in R (version 4.5.2) using the metafor package (22). Two-sided *P <* 0.05 was considered statistically significant for all tests except Cochran’s *Q*, for which *P <* 0.10 was used.

## 3 Results

### 3.1 Study selection

A total of 19,023 records were identified from five databases. After removing 17,171 duplicate records, the titles and abstracts of 1,852 unique records were screened. After excluding 1,614 records, the full text of the remaining 238 records was evaluated. An additional 219 records were excluded due to the following reasons: no extractable OR with 95% CI (n = 53), non-observational study design (n = 46), non-sleep exposure (n = 46), anxiety not the primary outcome (n = 38), disease-specific population (n = 18), overlapping sample (n = 1), and other reasons (n = 17). Hence, a total of 19 independent studies were included for the meta-analysis, with 21 effect-size records, given that two studies evaluated sleep quality and sleep duration. **Figure 1** shows the study selection process. The reasons for excluding studies are shown in **Supplementary Table 5**.

### 3.2 Study characteristics and quality assessment

**Table 1** describes the 19 studies which were included in this meta-analysis (25, 30, 33, 36, 37, 41). Published studies covered the period from 2014 to 2026, involving a total of 70,716 participants. Most of the included studies were conducted in China (15 studies, 79%), one in Canada, one in South Korea, one in India (29), and one in Israel (26). Of the included studies, 17 (89%) were cross-sectional and 2 studies employed a cohort design (24, 32). The size of the study sample ranged from 469 (38) to 14,417 participants (39).

**Table 1:** Characteristics of the included studies.

First author (year)	Country	Design	N	Exposure type	Tool (cutoff)	Anxiety scale (cutoff)	Covariates adjusted^e^	OR (95% CI)	QS^a^
*Sleep quality/disturbance*									
Potvin O (23)	Canada	CS	2,393	Sleep quality	PSQI (*>*5)	DSM-V/CIDI (Diagnosis)	A, S, Edu, Dep, Cog, Med, SH, CD, CVD	2.16 (1.30–3.60)	7
Kang HJ (24)	S. Korea	Cohort	1,204	Insomnia	Self-rated insomnia (≥3 nights/wk)	GMS-AGECAT (≥1)	A, S, U/R, Mar, Edu, Hous, Emp, Inc, SLE, SS, CD, PA, Dep, Ins, Cog, Alc	2.11 (1.57–2.82)	7b
Dong XL (25)	China	CS	758	Sleep quality	PSQI (*>*7)	SAS (≥50)	S, A, Edu, Econ, Cog, ADL, SS (stepwise)	6.23 (3.92–9.90)	7
Press Y (26)	Israel	CS	496	Sleep quality	Single-item (Dissatisfied)	SAST (≥24)	A, S, BMI, BI, Med	3.17 (1.71–5.88)	6
Tang R (27)	China	CS	1,132	Sleep quality	PSQI (*>*7)	HADS-A (*>*8)	A, Edu, Mar, Smk, Alc, PA	8.03 (3.53–18.28)	6
Shi WY (28)^c^	China	CS	3,897	Sleep quality	PSQI (≥7)	GAD-7 (≥5)	A, S, Liv, Mar, Inc, Edu, Occ, SS, Smk, Alc, BMI, CD, PM_2_._5_, Temp, Hum	5.12 (3.88–6.77)	9
Dahale AB (29)	India	CS	1,574	Insomnia	ISI (≥15)	PHQ-SADS (Threshold)	A, S, Liv, Mar, U/R, SES, Edu, Emp, CD, Dis, QoL, LS	5.98 (2.84–12.58)	6
Shen JL (30)	China	CS	7,158	Sleep quality	PSQI (≥6)	GAD-2 (≥3)	A, S, PA, Mar, Smk, Alc, Edu, Inc, BMI, SDur, Nap	4.41 (3.70–5.27)	7
Feng MY (31)	China	CS	4,582	Sleep quality	Single-item (Poor)	GAD-7 (≥5)	S, A, Mar, Edu, Dist, Fall, Vis, CD (+ clustering)	1.90 (1.59–2.28)	7
Liu T (32)^c^	China	Cohort	1,387	Sleep quality	Single-item (Poor)	GAD-7 (≥5)	S, A, Edu, Mar, Insur, Inc, Smk, Alc, PA	1.99 (1.17–3.39)	7b
Zhao XL (33)	China	CS	9,821	Sleep quality	PSQI (Binary)	SAS (≥50)	A, Edu, Inc, Chld, PA, SocAct, SS, Liv, ADL, SH, Nutr, Frail	1.89 (1.69–2.12)	9
Yuan YQ (34)	China	CS	2,497	Sleep quality	PSQI (Poor vs Good)	GAD-7 (≥5)	S, A, Mar, Edu, Inc, CD, SH	9.96 (7.34–13.52)	6
Zhang L (33)	China	CS	650	Sleep quality	PSQI (*>*7)	HAMA (≥14)	S, Alc, CD, PA (stepwise)	6.17 (3.72–10.23)	6
Xuan G (35)	China	CS	2,835	Insomnia	AIS (*>*3)	GAD-7 (≥5)	S, A, Edu, FHx-Dep, FHx-Dem, CHD, DM, DLP, Str, Cog, ADL, Det, Dep	25.73 (5.25–126.18)	7
Li Y (36)	China	CS	950	Sleep quality	PSQI (≥8)	GAD-7 (≥5)	S, A, Edu, CD, Smk, Alc	3.82 (2.83–5.16)	7
Wang X (37) *Sleep duration*	China	CS	1,643	Sleep quality	PSQI (*>*7)	GAD-7 (≥5)	PA, GI, Chld-Rel, ADL (forward LR)	4.61 (3.20–6.63)	7
He NF (38)	China	CS	469	Short duration	Single-item (*<*6h vs 7h)	SAS (≥50)	A, S, Mar, Edu, Liv, Econ	4.73 (2.15–10.43)	6
Shi WY (28)^c^	China	CS	3,897	Short duration	PSQI (≤6h vs 7h)	GAD-7 (≥5)	A, S, Liv, Mar, Inc, Edu, Occ, SS, Smk, Alc, BMI, CD, PM_2_._5_, Temp, Hum	2.09 (1.49–2.93)	9
Wang M (39)	China	CS	14,417	Short duration	Single-item (*<*7h vs 7–8h)	GAD-7 (≥5)	S, Reg, Econ, SH, LS, HC, Diet	2.07 (1.77–2.41)	10
Fan ZZ (40)^d^	China	CS	12,853	Short duration	Single-item (*<*7h vs 7–8h)	GAD-7 (≥5)	S, A, Mar, CD, Dis, Smk, Alc, PA, HC, Edu, Liv	2.25 (1.98–2.56)	10
Liu T (32)^c,d^	China	Cohort	1,387	Short duration	Single-item (*<*7h vs 7–9h)	GAD-7 (≥5)	S, A, Edu, Mar, Insur, Inc, Smk, Alc, PA	1.62 (1.10–2.40)	7b

CS, cross-sectional; QS, quality score; PSQI, Pittsburgh Sleep Quality Index; ISI, Insomnia Severity Index; AIS, Athens Insomnia Scale; GAD-7, Generalized Anxiety Disorder 7-item scale; GAD-2, 2-item version; SAS, Self-rating Anxiety Scale; HADS-A, Hospital Anxiety and Depression Scale–Anxiety subscale; HAMA, Hamilton Anxiety Rating Scale; SAST, Short Anxiety Screening Test; GMS-AGECAT, Geriatric Mental State–Automated Geriatric Examination for Computer Assisted Taxonomy; PHQ-SADS, Patient Health Questionnaire–Somatic, Anxiety and Depressive Symptoms.

^a^ Cross-sectional studies assessed using AHRQ criteria (0–11); cohort studies assessed using Newcastle-Ottawa Scale (0–9). Score ≥8 (AHRQ) or ≥7 (NOS) = high quality; 6–7 (AHRQ) or 4–6 (NOS) = moderate quality.

^b^ Cohort study assessed by Newcastle-Ottawa Scale (score out of 9).

^c^ Dual-exposure study contributing to both sleep quality and sleep duration subgroups.

^d^ Fan ZZ (40) and Liu T (32) used data from the Chinese Longitudinal Healthy Longevity Survey (CLHLS) with potential sample overlap; sensitivity analyses were performed (see **Table 4**).

^e^ Covariate: A, age; S, sex; Edu, education; Mar, marital status; Inc, income; Liv, living arrangement; Emp, employment; Occ, occupation; Hous, housing tenure; U/R, urban/rural; SES, socioeconomic status; Econ, economic status; Reg, region; Insur, insurance; Smk, smoking; Alc, alcohol; PA, physical activity/exercise; BMI, body mass index; CD, chronic diseases; CVD, cardiovascular diseases; CHD, coronary heart disease; DM, diabetes mellitus; DLP, dyslipidemia; Str, stroke; GI, gastrointestinal disease; Dep, depression; Cog, cognition/MMSE; ADL, activities of daily living; BI, Barthel Index; Dis, disability; Ins, insomnia; SDur, sleep duration; Nap, napping; SS, social support; SLE, stressful life events; SH, self-rated health; QoL, quality of life; LS, life satisfaction; Med, medication use; Nutr, nutritional status; Frail, frailty; Fall, falls; Vis, vision; Dist, distance to clinic; HC, healthcare access; Diet, dietary preference; SocAct, social activities; Chld, number of children; Chld-Rel, child relationship satisfaction; Det, overall deterioration; FHx-Dep, family history of depression; FHx-Dem, family history of dementia; Temp, temperature; Hum, humidity.

Of the 21 effect-size records, 16 were linked to sleep quality or sleep disturbance, and 5 linked to sleep duration. Two studies were classified into both the sleep exposure groups since they addressed multiple outcomes: Shi et al. (28) provided an effect size for short sleep duration and a separate effect size for poor sleep quality (PSQI), and Liu et al. (32) also addressed both sleep exposures. The Pittsburgh Sleep Quality Index (PSQI) was the sleep measure most frequently reported (*k* = 10). Insomnia-specific scales, including the Insomnia Severity Index, the Athens Insomnia Scale and single item self-reported insomnia were also reported (*k* = 3) as well as single item self-reported measures of sleep (*k* = 3). Cutoff scores for the PSQI varied among studies, with thresholds ranging from >5 to ≥6, ≥7, *>*7, or ≥8. For sleep duration, the original studies defined short sleep as <6, ≤6, or <7 hours per night relative to a reference category of 7–8 hours.

The most frequently used scale for assessing anxiety symptom frequency was the Generalized Anxiety Disorder 7-item (GAD-7) or Generalized Anxiety Disorder 2-item (GAD-2) scale (*k* = 10), followed by the Self-Rating Anxiety Scale (SAS; *k* = 3). Other anxiety symptom scales (*k* = 6) included the Hospital Anxiety and Depression Scale—Anxiety subscale (HADS-A), the Hamilton Anxiety Rating Scale (HAMA), and the Short Anxiety Screening Test (SAST) as well as diagnostic criteria for anxiety disorders. The methodological quality of the included cross-sectional studies was evaluated by AHRQ checklist and the quality of the included cohort studies was evaluated by the Newcastle-Ottawa Scale (NOS). A total of 6 (32%) studies were of high quality with AHRQ score ≥8 or NOS score ≥7 and 13 (68%) studies were of moderate quality with AHRQ score ranging from 6 to 7 or NOS score ranging from 4 to 6. None of the studies were of low quality. The full quality assessment is shown in **Supplementary Table 1**.

All 19 studies included in the systematic search provided adjusted odds ratios from multivariable models (**Table 1**). The majority of included studies controlled for age (*k* = 16) and sex (*k* = 16), with one study controlling for sex in a sample of only men (27). In addition to demographics, the majority of included studies controlled for variables such as education (*k* = 15), chronic conditions (*k* = 11), marital status (*k* = 10), and income/socioeconomic position (*k* = 10). Studies also controlled for smoking/alcohol use (*k* = 8) and physical activity (*k* = 8). Only three studies adjusted for depressive symptoms (23, 24, 35). The number of covariates in the different models varied between 4 and 16 (median 8). Please see Limitations for further discussion.

### 3.3 Main meta-analysis results

#### 3.3.1 Sleep quality/disturbance and anxiety

**Figure 2:**
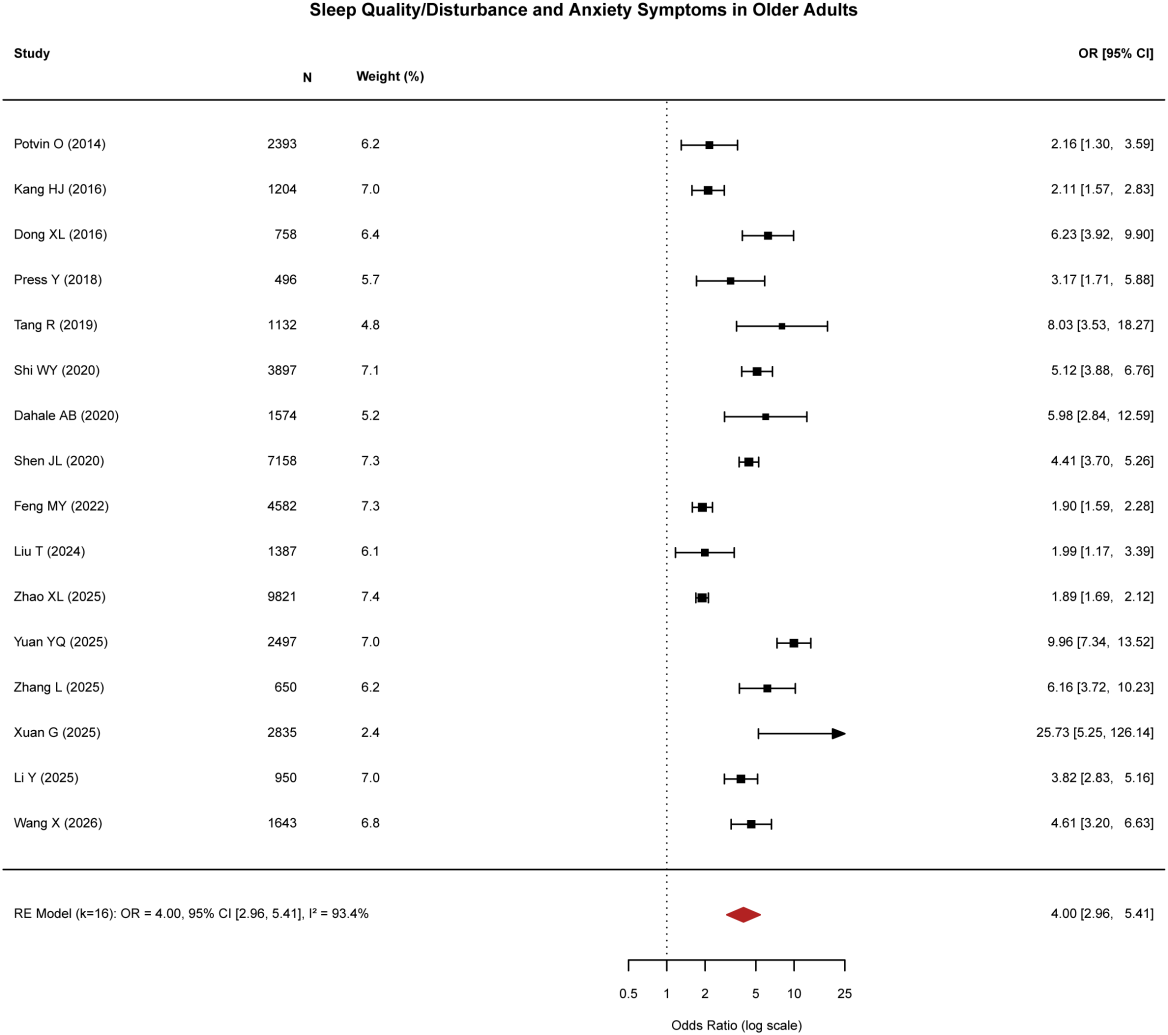
Forest plot of the association between poor sleep quality/disturbance and anxiety in older adults (*k* = 16). Pooled OR = 4.00 (95% CI: 2.96–5.41).

Pooling 16 studies (N = 42,977) that examined the association between poor sleep quality or disturbance and anxiety symptoms produced an OR of 4.00 (95% CI: 2.96–5.41, *P <* 0.001; **Figure 2**, **Table 2**). Heterogeneity was substantial (*I*^2^ = 93.4%, *Q* = 228.0, *P <* 0.001; *τ*^2^ = 0.315). The 95% prediction interval was wide (1.15 to 13.95), indicating that the magnitude of the association varied substantially across study settings, although the interval remained above the null. On the Galbraith plot, S123 (35) and S83 (34) stood out as the main outliers driving the observed heterogeneity (**Supplementary Figure 4**).

**Table 2:** Summary of main meta-analysis results.

Exposure	*k*	*N*	Pooled OR (95% CI)	*I*^2^ (%)	*Q* (*P*)	95% PI	FE OR (95% CI)
Sleep quality/disturbance	16	42,977	4.00 (2.96–5.41)	93.4	228.0 (*<*0.001)	1.15–13.95	2.89 (2.70–3.09)
Sleep duration	5	33,023	2.14 (1.85–2.46)	39.4	6.6 (0.158)	1.46–3.14	2.15 (1.97–2.36)

OR, odds ratio; CI, confidence interval; PI, prediction interval; FE, fixed-effect model. Pooled estimates derived using the DerSimonian–Laird random-effects model.

The fixed-effect model produced a smaller estimate (OR = 2.89, 95% CI: 2.70–3.09) in the same direction, reflecting the greater weight assigned to larger studies with more moderate effect sizes.

#### 3.3.2 Sleep duration and anxiety

**Figure 3:**
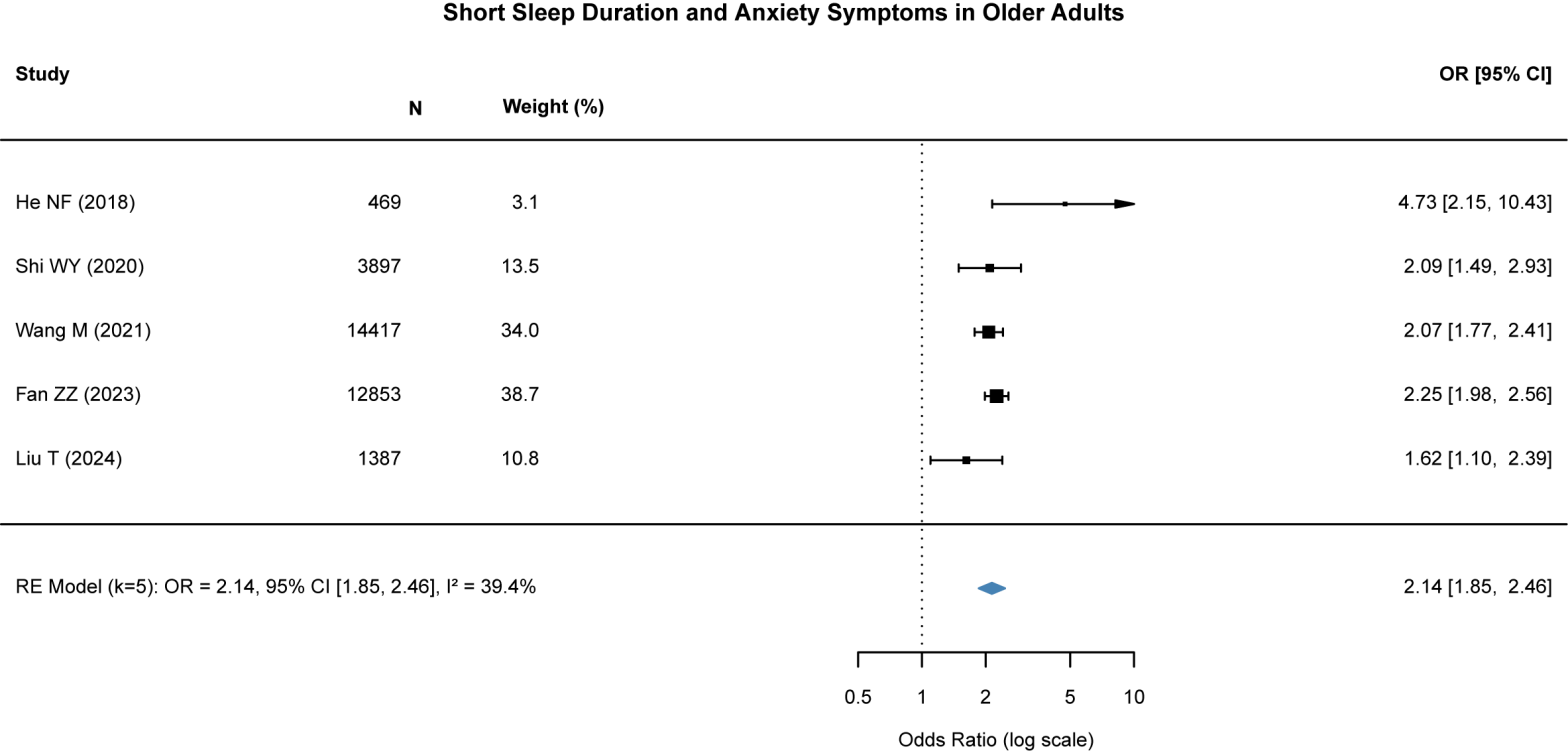
Forest plot of the association between short sleep and anxiety in older adults (*k* = 5). Pooled OR for this image was calculated using the DerSimonian–Laird random-effects model and equals 2.14 (95% CI: 1.85–2.46).

The combined results of the five studies (N = 33,023) examining short sleep duration and anxiety symptoms gave an overall OR of 2.14 (95% CI: 1.85–2.46, *P <* 0.001; **Figure 3**; **Table 2**). Between-study heterogeneity was low to moderate (*I*^2^ = 39.4%, *Q* = 6.6, *P* = 0.158; *τ*^2^ = 0.009). The 95% prediction interval was 1.46–3.14. The fixed-effect model gave an OR of 2.15 (95% CI: 1.97–2.36), nearly identical to the random-effects estimate. All included studies were based on Chinese populations, and three were based on the Chinese Longitudinal Healthy Longevity Survey (CLHLS) database (32, 39, 40). This overlap reduced the effective number of independent data sources, so CLHLS-specific sensitivity analyses were conducted.

#### 3.3.3 Comparison between exposure types

The pooled effect size for sleep quality/disturbance (OR = 4.00) was larger than that for sleep duration (OR = 2.14). A Wald *z*-test comparing these two independent pooled log-ORs showed a statistically significant difference (*z* = 3.69, *P <* 0.001), with a ratio of ORs of 1.87 (95% CI: 1.34–2.61). This comparison should be interpreted in the context of greater heterogeneity in the sleep quality/disturbance group (*I*^2^ = 93.4%) than in the sleep duration group (*I*^2^ = 39.4%), as well as differences in measurement depth, number of studies, and geographic coverage. Both sleep quality/disturbance and short sleep duration were significantly associated with anxiety.

### 3.4 Subgroup analysis and meta-regression

Subgroup analyses were conducted for the sleep quality/disturbance subgroup (*k* = 16) across five dimensions to identify potential sources of heterogeneity (**Table 3**; **Supplementary Figure 1**).

**Table 3:** Results of subgroup analyses and meta-regression for sleep quality/disturbance and anxiety symptoms.

Dimension	Subgroup	*k*	Pooled OR (95% CI)	*I*^2^ (%)	*P*_interaction_ ^a^
Country	China	12	4.45 (3.09–6.41)	95.0	0.261
	Non-China	4	2.78 (1.86–4.46)	59.6	
Study design	Cross-sectional	14	4.44 (3.18–6.22)	94.1	0.098
	Cohort	2	2.08 (1.61–2.69)	0.0	
Study quality	High (≥8)	4	2.54 (1.56–4.15)	92.9	0.058
	Moderate (6-7)	12	4.77 (3.34–6.81)	50.9	
Anxiety scale	GAD-7/GAD-2	8	4.36 (2.85–6.67)	93.8	0.825
	SAS	2	3.36 (1.05–10.79)	95.8	
	Other^b^	6	3.81 (2.36–6.13)	79.0	
Exposure tool	PSQI	10	4.59 (3.07–6.88)	95.1	0.227
	Insomnia scales^c^	3	5.54 (1.70–18.04)	86.4	
	Single-item	3	2.04 (1.63–2.56)	17.1	
Meta-regression					
Covariate			β	Q_model_ (P)	R^2^ (%)
Quality score		16	-0.239	2.32 (0.127)	11.1
Log (Sample sizxe)		16	-0.157	0.81 (0.367)	7.1
Publication year		16	0.032	0.56 (0.454)	0.0

^a^ *P*_interaction_ derived from meta-regression *Q*_model_ test with intercept (metafor package).

^b^ Other anxiety scales included DSM-V/CIDI, GMS-AGECAT, PHQ-SADS, HADS-A, HAMA, and SAST.

^c^ Insomnia scales included ISI, AIS, and self-rated insomnia.

Country. The pooled OR of Chinese studies (*k* = 12) was 4.45 (95% CI: 3.09–6.41, *I*^2^ = 95.0%) and the OR of non-Chinese studies (*k* = 4) was 2.78 (95% CI: 1.86–4.16, *I*^2^ = 59.6%). The between-subgroup difference did not reach statistical significance (*P*_interaction_ = 0.261).

Study design. Cross-sectional studies (*k* = 14) had a pooled OR of 4.44 (95% CI: 3.18–6.22, *I*^2^ = 94.1%), while the two cohort studies produced a lower estimate of 2.08 (95% CI: 1.61–2.69, *I*^2^ = 0.0%). The attenuation in cohort studies was apparent, but the between-subgroup difference did not reach statistical significance (*P*_interaction_ = 0.098).

Study quality. High-quality studies (*k* = 4, score ≥8) yielded a more conservative OR of 2.54 (95% CI: 1.56–4.15, *I*^2^ = 92.9%), whereas moderate-quality studies (*k* = 12) had a pooled OR of 4.77 (95% CI: 3.34–6.81, *I*^2^ = 90.9%). The difference approached but did not reach significance (*P*_interaction_ = 0.058).

Anxiety scale. The pooled OR for studies using GAD-7 or GAD-2 (*k* = 8) was 4.36 (95% CI: 2.85–6.67), for SAS (*k* = 2) was 3.36 (95% CI: 1.05–10.79), and for other instruments (*k* = 6) was 3.81 (95% CI: 2.36–6.13). No significant between-subgroup difference was found (*P*_interaction_ = 0.825).

Exposure measurement tool. PSQI-based studies (*k* = 10) had a pooled OR of 4.59 (95% CI: 3.07–6.88, *I*^2^ = 95.1%), studies using insomnia-specific scales (*k* = 3) had an OR of 5.54 (95% CI: 1.70–18.04, *I*^2^ = 86.4%), and those based on single-item measures (*k* = 3) had a lower OR of 2.04 (95% CI: 1.63–2.56, *I*^2^ = 17.1%). The between-subgroup difference was not statistically significant (*P*_interaction_ = 0.227).

Across all five subgroup dimensions, the pooled associations remained positive (ORs *>* 1). None of the between-subgroup tests was statistically significant. Cohort studies, high-quality studies, and single-item measures yielded more conservative but still statistically significant estimates.

Random-effects meta-regression with three covariates was performed. Study quality score (*β* = −0.239, *Q*_model_ = 2.32, *P* = 0.127, *R*^2^ = 11.1%), log-transformed sample size (*β* = −0.157, *Q*_model_ = 0.81, *P* = 0.367, *R*^2^ = 7.1%), and publication year (*β* = 0.032, *Q*_model_ = 0.56, *P* = 0.454, *R*^2^ = 0.0%) were each non-significant as individual predictors. Although study quality accounted for the largest share of explained variance, none of the included study-level variables explained the heterogeneity.

### 3.5 Sensitivity analysis

Several sensitivity analyses were carried out to check the robustness of the pooled estimates presented in **Table 4**.

**Table 4:** Results of sensitivity analyses.

Analysis	Subgroup	*k*	Pooled OR (95% CI)	*I*^2^ (%)
Primary analysis (reference)				
DL random-effects model	Sleep quality	16	4.00 (2.96–5.41)	93.4
DL random-effects model *Leave-one-out (sleep quality)*	Sleep duration	5	2.14 (1.85–2.46)	39.4
Range across 16 analyses *Excluding extreme OR values*	Sleep quality	15	3.68–4.26^a^	90.4–93.9
Exclude Xuan G (35)^b^	Sleep quality	15	3.82 (2.82–5.17)	93.7
Exclude Yuan YQ (34)^b^	Sleep quality	15	3.68 (2.79–4.86)	91.3
Exclude both *Fixed-effect model*	Sleep quality	14	3.52 (2.68–4.63)	91.5
Fixed-effect (Mantel–Haenszel)	Sleep quality	16	2.89 (2.70–3.09)	93.4
Fixed-effect (Mantel–Haenszel) *High-quality studies only (*≥*8)*	Sleep duration	5	2.15 (1.97–2.36)	39.4
High-quality only *CLHLS sample overlap*	Sleep quality	4	2.54 (1.56–4.15)	92.9
Keep Fan ZZ (40) only^c^	Sleep duration	3	2.35 (1.83–3.01)	43.8
Keep Liu T (32) only^c^ *Effect-size substitution*	Sleep duration	3	2.24 (1.42–3.52)	64.9
S57 incidence → prevalence OR^d^ *HKSJ correction*	Sleep quality	16	4.18 (3.07–5.69)	93.3
Hartung–Knapp–Sidik–Jonkman	Sleep quality	16	4.00 (2.86–5.60)	93.4
Hartung–Knapp–Sidik–Jonkman	Sleep duration	5	2.14 (1.69–2.71)	39.4

^a^ Minimum OR = 3.68 (excluding 34); maximum OR = 4.26 (excluding 31). All remained statistically significant.

^b^ Xuan G (35) OR = 25.73 and Yuan YQ (34) OR = 9.96 were the two highest effect sizes.

^c^ Wang M (39)Fan ZZ (40), and Liu T (32) used CLHLS data with potential sample overlap. Sensitivity analyses excluded overlapping studies, retaining one at a time.

^d^ Kang HJ (24) reported incidence OR from a cohort study; prevalence-equivalent OR = 3.78 substituted for sensitivity testing.

Leave-one-out analysis. The ORs obtained when each of the 16 sleep quality studies was removed in turn ranged from 3.68 (34) to 4.26 (31) and the *I*^2^ values ranged from 90.4% to 93.9% (**Supplementary Figure 2**). Removing any of them did not change the direction of the summary estimate, nor reduced it to non-significance.

Exclusion of extreme values. Removing the two with the largest effect sizes (35 (OR 25.73) and 34 (OR 9.96)) individually or in combination gave pooled ORs of 3.82 (95% CI: 2.82– 5.17), 3.68 (95% CI: 2.79–4.86) and 3.52 (95% CI: 2.68–4.63) all of which were highly significant. The outlier OR in the study of Xuan et al. (35) may have been due to the lower cut-off of the Athens Insomnia Scale (a score of >3 on a 0–24 scale rather than the conventional cut-off of ≥6) and the two-stage GAD screening procedure which may be more specific.

Fixed-effect model. The adjusted fixed-effect ORs for sleep quality and sleep duration were 2.89 (95% CI: 2.70 to 3.09) and 2.15 (95% CI: 1.97 to 2.36), respectively, and were consistent with the random-effects estimates.

High-quality studies only. When the analysis was limited to studies scoring ≥8 (*k* = 4), the pooled OR was 2.54 (95% CI: 1.56–4.15), reflecting a more conservative yet still significant association.

CLHLS overlap. For the sleep duration subgroup, retaining one CLHLS-derived estimate at a time preserved the positive association. The pooled estimate was OR = 2.35 (95% CI: 1.83–3.01, *k* = 3) when only Fan et al. (40) was retained among CLHLS-derived studies and OR = 2.24 (95% CI: 1.42–3.52, *k* = 3) when only Liu et al. (32) was retained.

After substituting the prevalence-equivalent OR of 3.78 with the incidence-based OR from Kang et al. (24), the pooled OR was 4.18 (95% CI: 3.07–5.69).

HKSJ correction. Applying the Hartung–Knapp–Sidik–Jonkman method widened the confidence intervals but preserved statistical significance. The estimated odds ratio was 4.00 (95% CI: 2.86–5.60) for sleep quality/disturbance and 2.14 (95% CI: 1.69–2.71) for sleep duration.

Cumulative meta-analysis. The cumulative OR from studies entered in increasing order of year of publication did not show any tendency to drift away from the approximate interval 3.0 to 4.0 as more were included, and remained close to this interval for 2020 and later publications with narrower confidence intervals (**Supplementary Figure 3**).

Across the eight sensitivity analyses, the direction and statistical significance of the associations were preserved.

### 3.6 Publication bias

**Figure 4:**
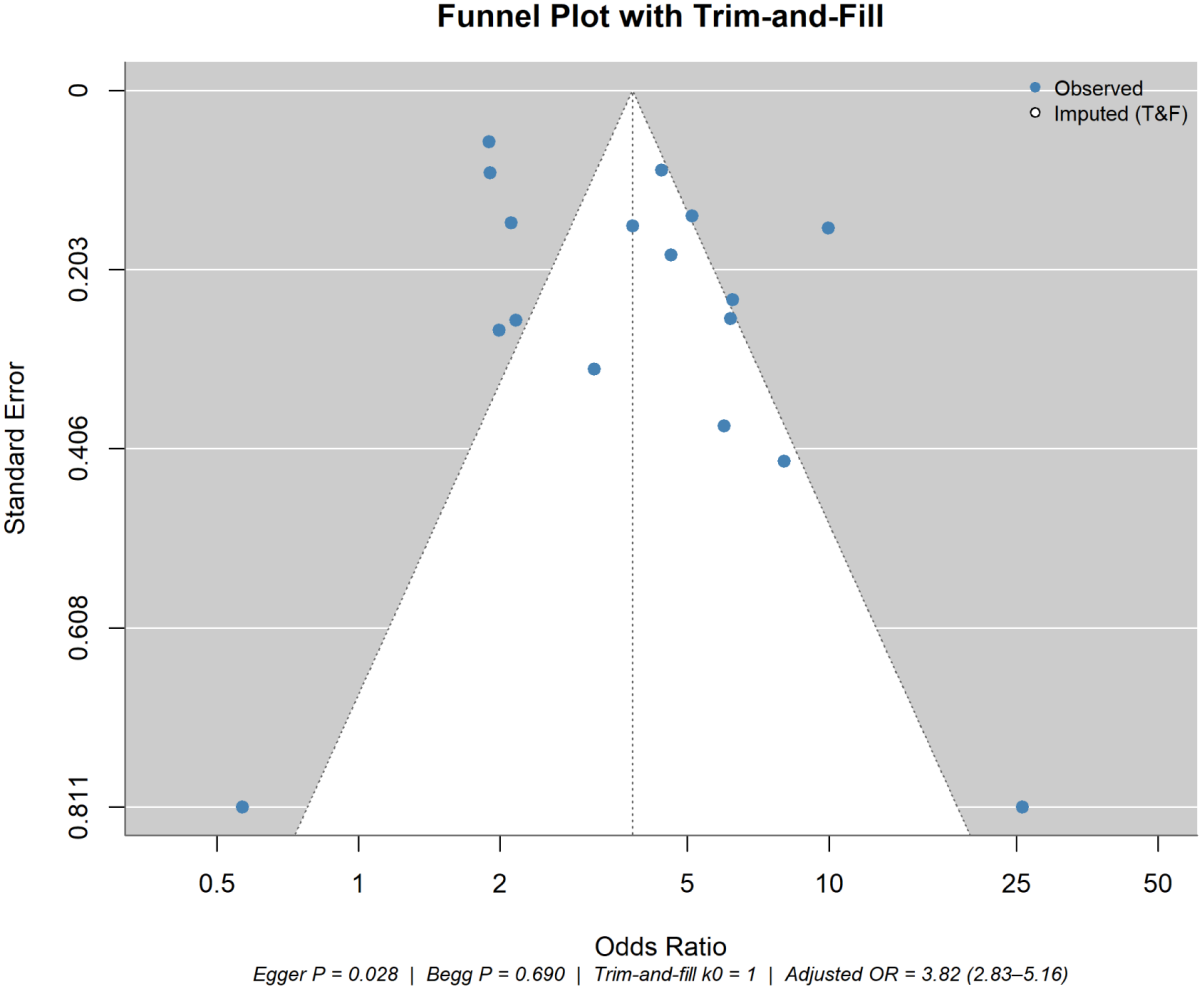
Funnel plot for sleep quality–anxiety. *k* = 16. The open circle is the study that was imputed by the trim–and–fill method, *k*_0_ =1.

As illustrated by the symmetry of the funnel plot in **Figure 4**, there is only a minor deviation towards the lower left corner suggesting that a few small studies with relatively small effects may have been left out. Egger’s regression test gives a statistically significant result (intercept = 0.622, *P* = 0.028). Begg’s rank correlation test gives a statistically non-significant result (*τ* = 0.083, *P* = 0.690) and provided no evidence of publication bias.

After applying the Duval and Tweedie trim-and-fill method, there was only one study on the left side of the funnel plot indicated by *k*_0_ = 1. The adjusted pooled OR after imputation was 3.82 (95% CI: 2.83–5.16). The OR did not change much from the original OR of 4.00 and it was still highly statistically significant. Although the Egger’s test was statistically significant with a high *I*^2^ of 93.4% this suggests that any heterogeneity is real and not due to publication bias. The trim and fill method imputed one study on the left of the plot with little effect on the pooled OR. The publication bias analysis for sleep duration was not conducted owing to the small number of studies (*k* = 5).

## 4 Discussion

This meta-analysis of 19 studies (*N* = 70,716) provides age-specific evidence that poor sleep quality is strongly associated with anxiety in older adults (OR = 4.00, 95% CI: 2.96–5.41, *k* = 16), while short sleep duration shows a moderate association (OR = 2.14, 95% CI: 1.85–2.46, *k* = 5). Both estimates are larger than those reported in several sleep–depression meta-analyses (OR = 2.60; 6), (RR = 1.92; 7), (RR = 1.27; 42), although differences between predominantly cross-sectional ORs and longitudinal RRs limit direct comparability. Bao et al. (7) found that persistent sleep disturbances predicted incident depression with RR = 3.90, approaching the present sleep quality– anxiety OR of 4.00. This pattern supports the relevance of chronic, multidimensional sleep disruption across late-life mental health outcomes.

### 4.1 Sleep quality versus duration: a novel dimensional comparison

The pooled association was larger for sleep quality/disturbance than for short sleep duration (OR = 4.00 vs. 2.14; OR ratio = 1.87, 95% CI: 1.34–2.61). This difference was statistically significant in the Wald *z*-test (*z* = 3.69, *P <* 0.001), but it should be interpreted as a dimensional comparison rather than a precise estimate of relative causal strength. The sleep quality/disturbance group included broader and more detailed instruments, whereas the sleep duration group relied mainly on categorical or single-item sleep-time measures and included fewer studies. Evidence from other populations supports this measurement pattern. In a 30-day daily diary study of 59 young adults, Chan et al. (43) found that sleep quality explained 15.9% of within-individual variability in next-day anxiety, compared with 7.7% for sleep duration. In 671 young adults, Muzni et al. (44) found that sleep quality was the strongest independent predictor of psychiatric morbidity (Cohen’s *f*^2^ = 0.033), whereas sleep duration did not predict mental health after controlling for sleep quality and chronotype (*f*^2^ = 0.001, *P* = 0.389). Similarly, Vaingankar et al. (45) reported in a multi-ethnic Asian population (*n* = 1,925) that sleep quality predicted positive mental health more strongly than sleep duration (*β* = 0.204 vs. *β* = 0.058).

Sleep must be considered in relation to other internal physiological states, most notably the neuroendocrine system that regulates the response to stress. van Dalfsen and Markus (46) systematically reviewed 17 experimental studies and concluded that the quality of sleep particularly influences HPA stress sensitivity whereas sleep quantity does not exert a significant influence. While five studies found that normal-range variation in sleep duration did not affect cortisol stress responses, studies using a variety of sleep quality indicators found that sleep disturbance selectively enhanced CRH-mediated cortisol reactivity (46). Cross-diagnostic evidence also supports this pattern: Bao et al. (7) found that long sleep onset latency—a quality indicator—was significantly associated with depression (OR = 1.33, 95% CI: 1.05–1.67), whereas short sleep duration (≤5 hours) was not (OR = 0.91, NS).

Although the subgroup analysis indicated no statistical difference (*P*_interaction_ = 0.227) between PSQI and insomnia-specific scales, the numerical pattern of stronger associations for PSQI-based studies (OR = 4.59) than single-item measures (OR = 2.04) is directionally consistent with the hypothesis that comprehensive, multidimensional sleep assessment better captures the sleep–anxiety relationship in older adult research and clinical practice.

### 4.2 Biological mechanisms

**Figure 5:**
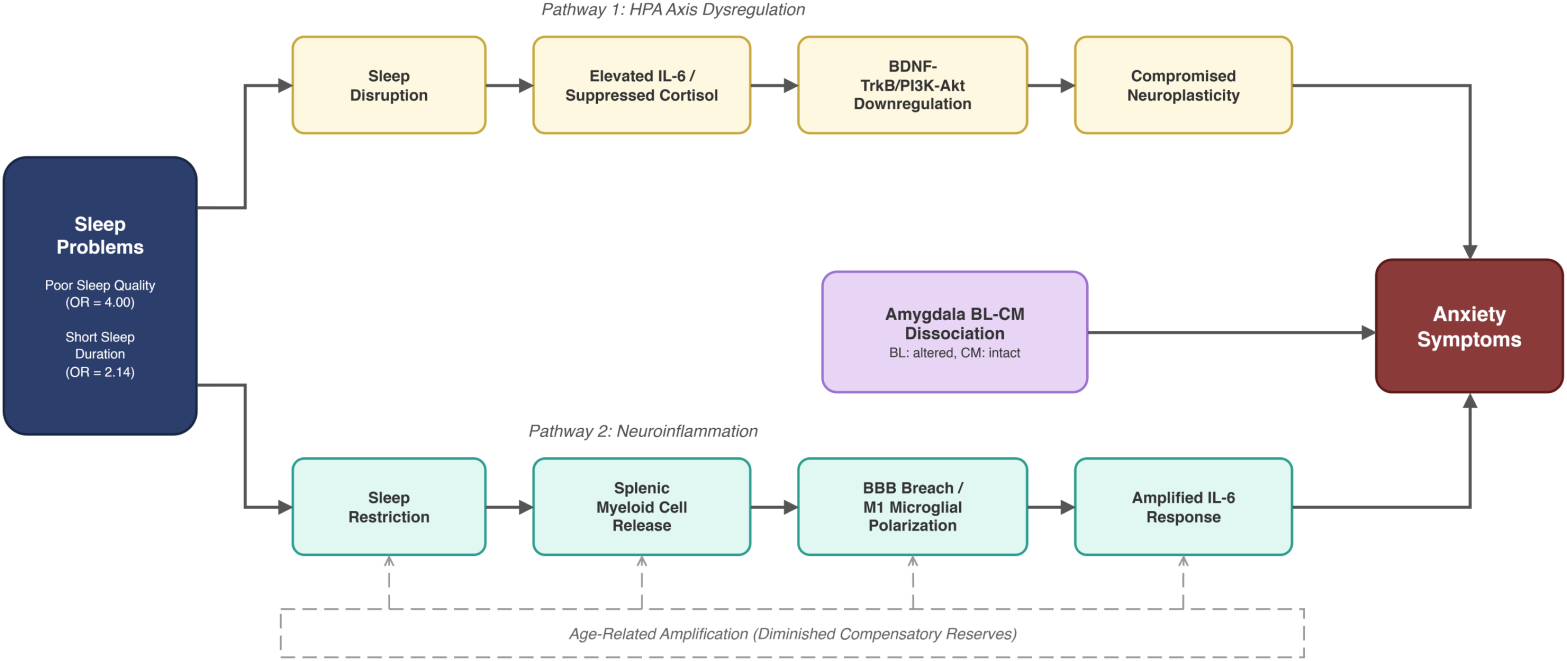
Dual-pathway mechanism by which sleep problems may contribute to anxiety in older adults. Pathway 1: HPA axis; Pathway 2: Neuroinflammation. Stressor processing further compromised by diminished age-related compensatory reserves.

The observed associations are biologically plausible through two pathways summarized in **Figure 5**.

The first pathway involves hypothalamic–pituitary–adrenal (HPA) axis dysregulation. Experimental evidence indicates that sleep disruption can increase stress reactivity. van Dalfsen and Markus (46) found that sleep quality selectively potentiates CRH-mediated cortisol stress reactivity without impairing negative feedback capacity. Cox and Olatunji (47) proposed that poor sleep quality leads to a blunted cortisol awakening response and dysregulated cortisol reactivity, resulting in elevated physiological arousal in the absence of objective threats. Direct experimental evidence documents this link: Wu et al. (48) demonstrated that four consecutive nights of sleep restriction produced a progressive dose–response increase in state anxiety (STAI: from 36.4 ± 7.1 to 47.4 ± 11.9) with a near-perfect correlation (*r* = 0.990, *P* = 0.001), accompanied by significant cortisol decline (*P <* 0.05). These findings support HPA-axis dysregulation as one plausible pathway linking poor sleep with anxiety.

Another pathway involves amygdala–prefrontal circuitry. Goldstein et al. (49) demonstrated that 24 hours of total sleep deprivation amplified bilateral amygdala anticipatory responses across threat conditions (*F* values *>* 10, *P <* 0.006), establishing a heightened threat expectancy state. Additionally, trait anxiety moderated the anterior insula response enhancement (*r* = 0.55, *P* = 0.02). Motomura et al. (50) demonstrated, using a within-subject crossover design (*n* = 18), that two days of experimentally induced sleep restriction significantly reduced left amygdala–mPFC functional connectivity (*P <* 0.001), and the reduction in REM sleep was correlated with left-hemisphere disconnection (*r* = 0.51, *P <* 0.05). This relationship is consistent with the mPFC’s inhibitory function on the amygdala and the HPA axis. Bao et al. (7) highlighted that age-related prefrontal cortex dysfunction can contribute to anxiety and physiological hyperarousal through disinhibition of the amygdala and HPA axis. These experimental paradigms involved acute or short-term sleep deprivation, often in younger adults; therefore, they support biological plausibility rather than causal proof in older adult populations.

Sahu et al. (51) proposed that these pathways may be exaggerated in older adults because sleep disruption can have a greater negative impact due to the decreased compensatory reserves related to neuroplasticity and immune function that occur with aging, rendering the elderly more vulnerable to the anxiogenic effects of sleep disturbance. Supporting this convergence, Rahmani et al. (52) synthesized evidence that chronic sleep deprivation drives hippocampal BDNF downregulation alongside elevated IL-1*β* and TNF-*α*, suggesting that the HPA axis and neuroinflammatory pathways may converge at the BDNF node to jointly predispose sleep-disturbed individuals to anxiety.

### 4.3 Evidence for directionality

The majority of the included studies (17 of 19) were cross-sectional, so the present meta-analysis supports associations rather than causal direction. Three external lines of evidence are consistent with a sleep-toanxiety pathway. First, experimental studies show that sleep loss can increase anxiety-relevant responses. In humans, Goldstein et al. (49) found that total sleep deprivation increased state anxiety from 30.29 to 39.47 (*T* = 4.83, *p* = 0.002), and Wu et al. (48) demonstrated a dose–response pattern over four nights of sleep restriction (*r* = 0.990, *P* = 0.001). In animal models, sleep deprivation induces anxiety-like behaviour (53, 54), with Xu et al. (54) confirming a causal chain in aged mice via splenectomy. A plausible biological mediator was identified by Irwin et al. (55), who showed that a single night of partial sleep deprivation activated inflammatory signalling, indexed by a marked increase in IL-6 expressing monocytes (*F* = 25.8, *P <* 0.001).

Second, longitudinal evidence: as noted in Section 4.1, Chan et al. (43) used a 30-day diary design and found that nightly sleep quality predicted next-day anxiety (*β* = −0.13, *p <* 0.001), whereas daytime anxiety did not predict subsequent sleep quality, indicating unidirectional temporality.

Third, intervention evidence: Belleville et al. (56) meta-analyzed 50 CBT-I trials (N = 2,690) and found that treating insomnia alone produced a moderate reduction in comorbid anxiety (*g* = 0.406, 95% CI: 0.318–0.493), with no additional benefit from adding anxiety management (*p* = 0.747). Lee and Harvey (57) confirmed this across 22 dCBT-I trials (N = 10,486; SMD = −0.29, *P <* 0.001). Taken together, experimental, longitudinal, and intervention evidence support a plausible sleep-to-anxiety pathway. The observational design of the included meta-analytic evidence, however, does not exclude bidirectionality or shared causes, including hyperarousal mechanisms (58).

### 4.4 Public health implications

Because the pooled ORs derive predominantly from cross-sectional data and the overall certainty of evidence was rated Very Low (GRADE), population-level causal claims are premature. Nonetheless, as a heuristic illustration, applying Levin’s formula (59) with the conservative high-quality-studies estimate (OR = 2.54) and the community prevalence of poor sleep quality among Chinese older adults of 41.5% (60) yields a PAF of 39.0%; for institutional settings where prevalence reaches 65% (61), the PAF rises to 50.0%. These figures should be interpreted as upper-bound projections rather than precise causal attributions, yet they underscore the potential preventive value of sleep interventions.

Given the strength of the sleep–anxiety association, non-pharmacological interventions should be prioritized. dCBT-I is a potentially effective approach: Lee and Harvey (57) showed that fully automated programmes that do not require a human therapist achieved significant anxiety reduction (SMD = −0.29, *P* = 0.001), making this approach suitable for resource-limited settings. Importantly, pharmacological alternatives such as benzodiazepines are inappropriate first-line options in older adults owing to risks of falls, cognitive impairment and drug dependency (62), further reinforcing the case for non-pharmacological sleep management as the preferred strategy.

### 4.5 Strengths and limitations

This meta-analysis focuses on the association between sleep and anxiety in adults aged ≥60 years. A second contribution is the direct dimensional comparison of sleep quality and sleep duration, two related but distinct sleep exposures. We employed HKSJ correction to guard against inflated significance in small meta-analyses, cumulative meta-analysis to assess temporal stability, and GRADE assessment to quantify evidence certainty. Using the trim-and-fill method, we found that only one missing study could be imputed with an adjusted OR of 3.82.

Several limitations define the interpretation of these findings. First, 17 of the 19 included studies were cross-sectional, which limits causal inference and leaves open bidirectional or shared-cause explanations. Second, 15 of 19 studies were conducted in China, and all sleep duration studies came from Chinese populations. Although the non-Chinese subgroup showed a consistent association (OR = 2.78, 95% CI: 1.86–4.16, *P*_interaction_ = 0.261), global generalizability remains limited. Third, between-study heterogeneity was high for sleep quality/disturbance (*I*^2^ = 93.4%). This heterogeneity reflects differences in PSQI cut-off scores (*>*5–≥8), anxiety measures, and the broad operational grouping of multidimensional sleep quality, insomnia-specific scales, clinical or self-reported insomnia, and single-item sleep problems. Fourth, only three studies (23, 24, 35) controlled for depressive comorbidity. Given the close relationship among depression, sleep disturbance, and anxiety symptoms, residual confounding by depressive comorbidity may have inflated the pooled ORs. Fifth, the sleep duration subgroup included only five studies, three of which were drawn from the CLHLS database, raising concerns about sample overlap despite consistent CLHLS sensitivity analyses. Short sleep thresholds also varied across the original studies (*<*6, ≤6, or <7 hours), and long sleep duration could not be analyzed because too few studies reported extractable ORs for this exposure. The GRADE certainty was Very Low, supporting the need for prospective cohorts with standardized sleep assessment and consistent adjustment for depressive symptoms, particularly in non-Chinese populations.

## 5 Conclusion

This systematic review and meta-analysis provides age-specific quantitative evidence on the association between sleep problems and anxiety symptoms in adults aged ≥60 years. Poor sleep quality/disturbance (OR = 4.00, 95% CI: 2.96–5.41) and short sleep duration (OR = 2.14, 95% CI: 1.85–2.46) were both significantly associated with anxiety. Sleep quality/disturbance showed a larger pooled association, although this comparison should be interpreted alongside its higher heterogeneity, broader measurement instruments, and the smaller evidence base for sleep duration.

These findings support the integration of standardized sleep quality assessment, particularly the PSQI, into routine geriatric health evaluations. The estimated population attributable fraction of 39% should be interpreted in light of the Very Low GRADE certainty rating and the predominance of cross-sectional evidence, but it indicates the potential public health relevance of sleep quality in community-dwelling older adults. Digital cognitive behavioral therapy for insomnia remains a plausible non-pharmacological option for older adults with co-occurring sleep and anxiety symptoms.

Prospective cohorts with adequate control for depressive comorbidity, standardized sleep assessment, and broader geographic representation are needed to clarify directionality and generalisability. Randomized trials of sleep interventions in older adults would further determine whether improving sleep reduces anxiety symptoms in this population.

## Data Availability

The original contributions presented in the study are included in the article/**Supplementary Material**. Further inquiries can be directed to the corresponding author.

## References

[1] United Nations, Department of Economic and Social Affairs, Population Division . World population ageing 2019: Highlights. ST/ESA/SER.A/444. New York: UN (2019).

[2] Miner B Kryger MH . Sleep in the aging population. Sleep Med Clinics. (2017) 12:31–8. doi:10.1016/j.jsmc.2016.10.008. PMID: 28159095 PMC5300306

[3] Gulia KK Kumar VM . Sleep disorders in the elderly: a growing challenge. Psychogeriatrics. (2018) 18:155–65. doi:10.1111/psyg.12319. PMID: 29878472

[4] Wolitzky-Taylor KB Castriotta N Lenze EJ Stanley MA Craske MG . Anxiety disorders in older adults: a comprehensive review. Depression Anxiety. (2010) 27:190–211. doi:10.1002/da.20653. PMID: 20099273

[5] Ben Simon E Rossi A Harvey AG Walker MP . Overanxious and underslept. Nat Hum Behav. (2020) 4:100–10. doi:10.1038/s41562-019-0754-8. PMID: 31685950

[6] Baglioni C Battagliese G Feige B Spiegelhalder K Nissen C Voderholzer U . Insomnia as a predictor of depression: a meta-analytic evaluation of longitudinal epidemiological studies. J Affect Disord. (2011) 135:10–9. doi:10.1016/j.jad.2011.01.011. PMID: 21300408

[7] Bao Y-P Han Y Ma J Wang R-J Shi L Wang T-Y . Cooccurrence and bidirectional prediction of sleep disturbances and depression in older adults: meta-analysis and systematic review. Neurosci Biobehav Rev. (2017) 75:257–73. doi:10.1016/j.neubiorev.2017.01.032. PMID: 28179129

[8] Page MJ McKenzie JE Bossuyt PM Boutron I Hoffmann TC Mulrow CD . The PRISMA 2020 statement: an updated guideline for reporting systematic reviews. BMJ. (2021) 372:n71. doi:10.1136/bmj.n71. PMID: 33782057 PMC8005924

[9] Stroup DF Berlin JA Morton SC Olkin I Williamson GD Rennie D . Metaanalysis of observational studies in epidemiology: a proposal for reporting. JAMA. (2000) 283:2008–12. doi:10.1001/jama.283.15.2008. PMID: 10789670

[10] Haddaway NR Page MJ Pritchard CC McGuinness LA . PRISMA2020: an R package and Shiny app for producing PRISMA 2020-compliant flow diagrams, with interactivity for optimised digital transparency and Open Synthesis. Campbell Syst Rev. (2022) 18:e1230. doi:10.1002/cl2.1230. PMID: 36911350 PMC8958186

[11] Buysse DJ . Sleep health: can we define it? Does it matter? Sleep. (2014) 37:9–17. doi:10.5665/sleep.3298. PMID: 24470692 PMC3902880

[12] Rostom A Dubé C Cranney A Saloojee N Sy R Garritty C . Celiac disease. evidence reports/technology assessments, no. 104. In: Agency for healthcare research and quality (US) appendix D. Quality assessment forms. AHRQ publication no. 04-E029-2. Rockville, MD: Agency for Healthcare Research and Quality (US) (2004).

[13] Wells GA Shea B O’Connell D Peterson J Welch V Losos M . The Newcastle-Ottawa Scale (NOS) for assessing the quality of nonrandomised studies in meta-analyses (2000). Available online at: http://www.ohri.ca/programs/clinical_epidemiology/oxford.asp (Accessed January 15, 2026).

[14] DerSimonian R Laird N . Meta-analysis in clinical trials. Controlled Clin Trials. (1986) 7:177–88. doi:10.1016/0197-2456(86)90046-2. PMID: 3802833

[15] Higgins JPT Thompson SG Deeks JJ Altman DG . Measuring inconsistency in meta-analyses. BMJ. (2003) 327:557–60. doi:10.1136/bmj.327.7414.557. PMID: 12958120 PMC192859

[16] IntHout J Ioannidis JPA Rovers MM Goeman JJ . Plea for routinely presenting prediction intervals in meta-analysis. BMJ Open. (2016) 6:e010247. doi:10.1136/bmjopen-2015-010247. PMID: 27406637 PMC4947751

[17] Hartung J Knapp G . A refined method for the meta-analysis of controlled clinical trials with binary outcome. Stat Med. (2001) 20:3875–89. doi:10.1002/sim.1009. PMID: 11782040

[18] Egger M Davey Smith G Schneider M Minder C . Bias in meta-analysis detected by a simple, graphical test. BMJ. (1997) 315:629–34. doi:10.1136/bmj.315.7109.629. PMID: 9310563 PMC2127453

[19] Begg CB Mazumdar M . Operating characteristics of a rank correlation test for publication bias. Biometrics. (1994) 50:1088–101. doi:10.2307/2533446 7786990

[20] Duval S Tweedie R . Trim and fill: a simple funnel-plot-based method of testing and adjusting for publication bias in meta-analysis. Biometrics. (2000) 56:455–63. doi:10.1111/j.0006-341X.2000.00455.x. PMID: 10877304

[21] Guyatt GH Oxman AD Schunemann HJ Tugwell P Knottnerus A . GRADE guidelines: a new series of articles in the Journal of Clinical Epidemiology. J Clin Epidemiol. (2011) 64:380–2. doi:10.1016/j.jclinepi.2010.09.011. PMID: 21185693

[22] Viechtbauer W . Conducting meta-analyses in R with the metafor package. J Stat Software. (2010) 36:1–48. doi:10.18637/jss.v036.i03

[23] Potvin O Lorrain D Belleville G Grenier S Preville M . Subjective sleep characteristics associated with anxiety and depression in older adults: a population-based study. Int J Geriatric Psychiatry. (2014) 29:1262–70. doi:10.1002/gps.4106. PMID: 24733621

[24] Kang H-J Bae K-Y Kim S-W Shin I-S Yoon J-S Kim J-M . Anxiety symptoms in korean elderly individuals: a two-year longitudinal community study. Int Psychogeriatrics. (2016) 28:423–33. doi:10.1017/S1041610215001301. PMID: 26299311

[25] Dong X Sun W Yuan Y . Analysis of anxiety status and influencing factors among community-dwelling older adults in Jiangxi province. Modern Prev Med. (2016) 43:2378–81.

[26] Press Y Punchik B Freud T . The association between subjectively impaired sleep and symptoms of depression and anxiety in a frail elderly population. Aging Clin Exp Res. (2018) 30:755–65. doi:10.1007/s40520-017-0837-1. PMID: 29022191

[27] Tang R Yang SS Ma XN Wang JH Liu M Wang SS . Analysis of sleep factors affecting anxiety and depression in elderly male physical examination population. Chin J Clin Healthcare. (2019) 22:161–5. doi:10.3969/J.issn.1672-6790.2019.02.005

[28] Shi WY Guo MH Du P Zhang Y Wang JN Li TT . Association of sleep with anxiety in the elderly aged 60 years and older in China. Zhonghua liu xing bing xue za zhi = Zhonghua liuxingbingxue zazhi. (2020) 41:13–9. doi:10.3760/cma.j.issn.0254-6450.2020.01.004. PMID: 32062936

[29] Dahale AB Jaisoorya TS Manoj L Kumar GS Gokul GR Radhakrishnan R . Insomnia among elderly primary care patients in India. primary Care companion For CNS Disord. (2020) 22:25200. doi:10.4088/PCC.19m02581. PMID: 32441494

[30] Shen J Zhang H Wang Y Abdulai T Niu M Luo Z . Dose-response association of sleep quality with anxiety symptoms in chinese rural population: the henan rural cohort. BMC Public Health. (2020) 20:1297. doi:10.1186/s12889-020-09400-2. PMID: 32854672 PMC7450150

[31] Feng M Xu T Han H . Analysis of anxiety status and influencing factors among rural older adults in western China. Chin J Health Educ. (2022) 38:173–6. doi:10.16168/j.cnki.issn.1002-9982.2022.02.016

[32] Liu T Li C Zhao M Xi B . Effects of sleep status on depression and anxiety among chinese older adults based on CLHLS data. Prev Med Tribune. (2024) 30:487–93. doi:10.16406/j.pmt.issn.1672-9153.2024.7.02. PMID: 41867606

[33] Zhang L Gao X Li J Huang Y Liu X Ma R . Analysis of the correlation between severity of anxiety and depressive symptoms and sleep quality in older adults. South China J Prev Med. (2025) 51:230–3.

[34] Yuan Y Huang W Hu C Zhang W . The interaction of physical activity and sleep quality with depression and anxiety in older adults. Front Public Health. (2025) 13:1674459. doi:10.3389/fpubh.2025.1674459. PMID: 41211411 PMC12588915

[35] Xuan G Ding Y Zhu H Ma N Li XH . Current status and influencing factors of depressive and anxiety symptoms among older adults in a district of Shanghai. Med Inf. (2025) 38:86–91. doi:10.3969/j.issn.1006-1959.2025.04.014

[36] Li Y Chen Y Wang H Wang X Li M Ding H . Interaction analysis of sleep disorders and frailty on anxiety in rural older adults. J Fujian Med Univ (Social Sci Edition). (2025) 26:33–8.

[37] Wang X Xu WY Xiong JX Li C Cai J Wang ZW . Current status and related factors of depressive and anxiety symptoms among community-dwelling older adults in Shanghai. Chin J Psychiatry. (2026) 59:30–8. doi:10.3760/cma.j.cn113661-20250425-00191. PMID: 30704229

[38] He NF Yue MJ Zhao YK Li HH Lu YX Wu FY . Correlation between nighttime sleep duration and anxiety in retired older adults. Chin J Gerontology. (2018) 38:213–5. doi:10.3969/j.issn.1005-9202.2018.01.090

[39] Wang M Pan Q . Urban-rural differences and influencing factors of anxiety status among Chinese older adults. Chin Gen Pract. (2021) 24:3963–70. doi:10.12114/j.issn.1007-9572.2021.00.294

[40] Fan Z Wang J Tan P . Association between sleep status and risk of anxiety disorder among Chinese older adults. Med Soc. (2023) 36:85–9. doi:10.13723/j.yxysh.2023.09.015

[41] Zhao X Yuan X Meng D Liang H Xiong Y Li Y . Prevalence and correlates of anxiety and depression among chronically ill older adults in zunyi, China: a cross-sectional study. Front Psychol. (2025) 16:1560650. doi:10.3389/fpsyg.2025.1560650. PMID: 40248831 PMC12004976

[42] Li X Huang L Luo Y Huang Q Lan Y Xia Y . Nighttime sleep duration and risk of depression among middle-aged and older adults: dose-response meta-analysis. Front Physiol. (2023) 14:1085091. doi:10.3389/fphys.2023.1085091. PMID: 36935736 PMC10017495

[43] Chan WS Lam SCY Ng ASY Lobo S . Daily associations of sleep quality and sleep duration with anxiety in young adults: the moderating effect of alexithymia. Behav Sleep Med. (2022) 20:787–97. doi:10.1080/15402002.2021.2016406. PMID: 34927498

[44] Muzni K Groeger JA Dijk D-J Lazar AS . Self-reported sleep quality is more closely associated with mental and physical health than chronotype and sleep duration in young adults: a multi-instrument analysis. J Sleep Res. (2021) 30:e13152. doi:10.1111/jsr.13152. PMID: 32783404 PMC11475679

[45] Vaingankar JA Muller-Riemenschneider F Chu AHY Subramaniam M Tan LWL Chong SA . Sleep duration, sleep quality and physical activity, but not sedentary behaviour, are associated with positive mental health in a multi-ethnic Asian population: a crosssectional evaluation. Int J Environ Res Public Health. (2020) 17:8489. doi:10.3390/ijerph17228489. PMID: 33207763 PMC7697582

[46] van Dalfsen JH Markus CR . The influence of sleep on human hypothalamic–pituitary–adrenal (HPA) axis reactivity: a systematic review. Sleep Med Rev. (2018) 39:187–94. doi:10.1016/j.smrv.2017.10.002. PMID: 29126903

[47] Cox RC Olatunji BO . A systematic review of sleep disturbance in anxiety and related disorders. J Anxiety Disord. (2016) 37:104–29. doi:10.1016/j.janxdis.2015.12.001. PMID: 26745517

[48] Wu H Zhao Z Stone WS Huang L Zhuang J He B . Effects of sleep restriction periods on serum cortisol levels in healthy men. Brain Res Bull. (2008) 77:241–5. doi:10.1016/j.brainresbull.2008.07.013. PMID: 18761394

[49] Goldstein AN Greer SM Saletin JM Harvey AG Nitschke JB Walker MP . Tired and apprehensive: anxiety amplifies the impact of sleep loss on aversive brain anticipation. J Neurosci. (2013) 33:10607–15. doi:10.1523/JNEUROSCI.5578-12.2013. PMID: 23804084 PMC3693050

[50] Motomura Y Kitamura S Enomoto M Kamei Y Inagaki N Mishima K . Two days’ sleep debt causes mood decline during resting state via diminished amygdala–prefrontal connectivity. Sleep. (2017) 40:zsx133. doi:10.1093/sleep/zsx133. PMID: 28977527

[51] Sahu M Tripathi R Jha NK Jha SK . Cross talk mechanism of disturbed sleep patterns in neurological and psychological disorders. Neurosci Biobehav Rev. (2022) 140:104767. doi:10.1016/j.neubiorev.2022.104767. PMID: 35811007

[52] Rahmani M Rahmani F Rezaei N . The brain-derived neurotrophic factor: missing link between sleep deprivation, insomnia, and depression. Neurochem Res. (2020) 45:221–31. doi:10.1007/s11064-019-02914-1. PMID: 31782101

[53] Manchanda S Mishra R Singh R Iber M Thinschmidt JS . Low-grade neuroinflammation due to chronic sleep deprivation results in anxiety and learning and memory impairments. Mol Cell Biochem. (2018) 449:63–72. doi:10.1007/s11010-018-3343-7. PMID: 29549603

[54] Xu Q Xu X-H Bhatt S Bhatt SR Bhatt S . Sleep restriction induces changes in inflammation and anxiety-like behavior via the spleen-brain axis. Aging (Albany NY). (2020) 12:6310–26. doi:10.18632/aging.103659. PMID: 32741775 PMC7467362

[55] Irwin MR Carrillo C Olmstead R . Sleep loss activates cellular markers of inflammation: sex differences. Brain Behavior Immun. (2010) 24:54–7. doi:10.1016/j.bbi.2009.06.001. PMID: 19520155 PMC2787978

[56] Belleville G Cousineau H Levrier K St-Pierre-Delorme M-È . Meta-analytic review of the impact of cognitive-behavior therapy for insomnia on concomitant anxiety. Clin Psychol Rev. (2011) 31:638–52. doi:10.1016/j.cpr.2011.02.004. PMID: 21482322

[57] Lee S Oh JW Park KM Lee S Lee E . Digital cognitive behavioral therapy for insomnia on depression and anxiety: a systematic review and meta-analysis. npj Digital Medicine. (2023) 6(1):52. doi: 10.1038/s41746-023-00800-3. PMID: 36966184 PMC10039857

[58] Magee JC Carmin CN . The relationship between sleep and anxiety in older adults. Curr Psychiatry Rep. (2010) 12:13–9. doi:10.1007/s11920-009-0087-9. PMID: 20425305

[59] Levin ML . The occurrence of lung cancer in man. Acta Unio Int Contra Cancrum. (1953) 9:531–41. 13124110

[60] Luo J Zhu G Zhao Q Guo Q Meng H Hong Z . Prevalence and risk factors of poor sleep quality among Chinese elderly in an urban community: results from the Shanghai aging study. PloS One. (2013) 8:e81261. doi:10.1371/journal.pone.0081261. PMID: 24282576 PMC3839883

[61] Pangaribuan DH Liang H-W Liao H-J Lu L-C Wu Y-T Wang T-Y . Global occurrence rates of sleep disturbances among institutionalized older adults: a systematic review and meta-analysis. Sleep Med Rev. (2025) 79:102091. doi:10.1016/j.smrv.2025.102091. PMID: 40239318

[62] American Geriatrics Society . American Geriatrics Society 2023 updated AGS Beers Criteria for potentially inappropriate medication use in older adults. J Am Geriatrics Soc. (2023) 71:2052–81. doi:10.1111/jgs.18372. PMID: 37139824 PMC12478568

