# Supplementary material for: Association of sleep quality and sleep duration with anxiety symptoms in older adults: a systematic review and meta-analysis
Source: Front Psychiatry. 2026 May 28;17:1838523. doi: 10.3389/fpsyt.2026.1838523 (PMC13253953; doi:10.3389/fpsyt.2026.1838523)
Supplement: Supplementary file 1 [file SupplementaryFile1.pdf]

Supplementary Material: Association of Sleep Quality and Sleep Duration with Anxiety Symptoms in Older Adults: A Systematic Review and Meta-Analysis

Junjie Zhang [1,†], Xiaochun Zhu [1,†], Lei Zhang [1], Yanmin Shen [1], Meili Shi [1] and Haiying Min [1,*]

[1] *Clinical Research Center for Mental Disorders, Shanghai Pudong New Area Mental Health Center, School of Medicine, Tongji University, Shanghai, China*

Correspondence*:
Haiying Min
446152628@qq.com

TABLE S1. QUALITY ASSESSMENT OF INCLUDED STUDIES

A. Cross-Sectional Studies — AHRQ Checklist (0–11)

Study	Q1	Q2	Q3	Q4	Q5	Q6	Q7	Q8	Q9	Q10	Q11	Total	Grade
Potvin (2014)[S36]	Y	Y	Y	Y	Y	N	Y	NR	N	N	Y	7	Moderate
Dong (2016)[R5]	Y	Y	Y	Y	Y	N	Y	NR	N	N	Y	7	Moderate
He (2018)[U1]	Y	Y	Y	Y	N	N	Y	NR	N	N	Y	6	Moderate
Press (2018)[S95]	Y	Y	Y	Y	N	N	Y	NR	N	N	Y	6	Moderate
Tang (2019)[U2]	Y	Y	Y	Y	N	N	Y	NR	N	N	Y	6	Moderate
Dahale (2020)[S66]	Y	Y	Y	Y	N	N	Y	NR	N	N	Y	6	Moderate
Shi (2020)[S32]	Y	Y	Y	Y	Y	Y	Y	Y	N	N	Y	9	High
Shen (2020)[S97]	Y	Y	Y	Y	Y	N	Y	NR	N	N	Y	7	Moderate
Wang M (2021)[S151]	Y	Y	Y	Y	Y	Y	Y	Y	Y	N	Y	10	High
Feng (2022)[S148]	Y	Y	Y	Y	Y	N	Y	NR	N	N	Y	7	Moderate
Fan (2023)[S131]	Y	Y	Y	Y	Y	Y	Y	Y	Y	N	Y	10	High
Yuan (2025)[S83]	Y	Y	Y	Y	N	N	Y	NR	N	N	Y	6	Moderate
Zhao (2025)[S75]	Y	Y	Y	Y	Y	Y	Y	Y	N	N	Y	9	High
Xuan (2025)[S123]	Y	Y	Y	Y	Y	N	Y	NR	N	N	Y	7	Moderate
Zhang L (2025)[S122]	Y	Y	Y	Y	N	N	Y	NR	N	N	Y	6	Moderate
Li Y (2025)[R7]	Y	Y	Y	Y	Y	N	Y	NR	N	N	Y	7	Moderate
Wang X (2026)[U3]	Y	Y	Y	Y	Y	N	Y	NR	N	N	Y	7	Moderate

AHRQ Cross-Sectional Study Quality Assessment Tool items: Q1 = source of information clearly defined; Q2 = eligibility criteria listed; Q3 = study time period identified; Q4 = participants recruited in consecutive or random manner; Q5 = evaluators of subjective components masked to other aspects; Q6 = description of quality assurance procedures; Q7 = dependent/independent variables defined; Q8 = confounders and interactions assessed; Q9 = handling of missing data described; Q10 = response rate and completeness reported; Q11 = follow-up results reported (or cross-sectional analysis completed).
Y = Yes, N = No, NR = Not Reported. High: ≥8; Moderate: 6–7; Low: <6.

B. Cohort Studies — Newcastle–Ottawa Scale (0–9)

Study	S1	S2	S3	S4	C1	C2	O1	O2	Total	Grade
Kang (2016)[S57]	⋆	⋆	⋆	—	⋆	⋆	⋆	⋆	7	High
Liu T (2024)[S130]	⋆	⋆	⋆	—	⋆	⋆	⋆	⋆	7	High

NOS domains: Selection (S1–S4): representativeness of exposed cohort, selection of non-exposed, ascertainment of exposure, outcome not present at start; Comparability (C1–C2): comparability on basis of design or analysis; Outcome (O1–O2): assessment of outcome, adequacy of follow-up.
⋆ = criterion met; — = criterion not met. High: $\geq$7; Moderate: 4–6; Low: $\leq$3.

TABLE S2. PRISMA 2020 CHECKLIST

No.	Item	Checklist Item	Location
**TITLE**			
1	Title	Identify the report as a systematic review, meta-analysis, or both.	Title page
**ABSTRACT**			
2	Abstract	Provide a structured summary including background, methods, results, and conclusion.	Abstract
**INTRODUCTION**			
3	Rationale	Describe the rationale for the review in the context of existing evidence.	Introduction
4	Objectives	Provide an explicit statement of the objective(s) of the review.	Introduction
**METHODS**			
5	Eligibility criteria	Specify eligibility criteria and information sources.	Methods
6	Information sources	Specify all databases and date of last search.	Methods
7	Search strategy	Present the complete search strategy for at least one database (see Table S4).	Supplementary Table S4
8	Selection process	Describe the study selection process.	Methods
9	Data collection process	Describe the method of data extraction.	Methods
10a	Data items	List and define all outcomes for which data were sought.	Methods
10b	Data items	List and define all other variables for which data were sought.	Methods
11	Study risk of bias	Describe the methods used for assessing risk of bias of included studies.	Methods
12	Effect measures	Specify the effect measure(s) used in the synthesis.	Methods
13a	Synthesis methods	Describe the processes used to decide which studies were eligible for each synthesis.	Methods
13b	Synthesis methods	Describe any methods required to prepare data for synthesis.	Methods
13c	Synthesis methods	Describe any methods used to tabulate or visually display results.	Methods
13d	Synthesis methods	Describe any methods used to synthesize results.	Methods
13e	Synthesis methods	Describe any sensitivity analyses conducted.	Methods
13f	Synthesis methods	Describe any methods used to assess heterogeneity.	Methods
14	Reporting bias assessment	Describe any methods used to assess reporting or publication bias.	Methods
15	Certainty assessment	Describe any methods used to assess certainty of evidence (e.g., GRADE).	Methods; Supplementary Table S3
**RESULTS**			

Continued on next page

No.	Item	Checklist Item	Location
16a	Study selection	Describe the results of the search and selection process (see Figure 1).	Results; Figure 1
16b	Study selection	Cite excluded studies and reasons (see Table S5).	Supplementary Table S5
17	Study characteristics	Cite each included study and present its characteristics (see Table 1).	Results; Table 1
18	Risk of bias in studies	Present assessments of risk of bias for each included study (see Table S1).	Supplementary Table S1
19	Results of individual studies	Present results for all individual studies (see Figures 2–3).	Results; Figures 2–3
20a	Results of syntheses	For each synthesis, briefly summarize the characteristics and risk of bias (see Table 2).	Results; Table 2
20b	Results of syntheses	Present results of each meta-analysis, including CIs and heterogeneity.	Results; Table 2
20c	Results of syntheses	Present results of all investigations of heterogeneity (see Table 3).	Results; Table 3
20d	Results of syntheses	Present results of all sensitivity analyses (see Table 4).	Results; Table 4
21	Reporting biases	Present results of assessment of reporting or publication bias.	Results
22	Certainty of evidence	Present assessments of certainty of evidence (see Table S3).	Supplementary Table S3
**DISCUSSION**			
23a	Discussion	Provide a general interpretation of the results.	Discussion
23b	Discussion	Discuss any limitations of the evidence and of the review process.	Discussion
23c	Discussion	Discuss implications of the results.	Discussion
23d	Discussion	Discuss the generalizability of the findings.	Discussion
24	Registration and protocol	Provide registration information and indicate amendments.	Methods
**OTHER INFORMATION**			
25	Support	Describe sources of financial and non-financial support.	Funding
26	Competing interests	Declare any competing interests of review authors.	Conflict of Interest
27	Availability of data	Report which of the data, code, and materials are available.	Data Availability Statement

TABLE S3. GRADE EVIDENCE PROFILE

Outcome	k	Risk of Bias	Inconsistency	Indirectness	Imprecision	Publication Bias	Quality	OR (95% CI)
Sleep quality → Anxiety	16	No downgrade[a]	↓1[b]	No downgrade[c]	No downgrade[d]	No downgrade[e]	**Very Low**[f]	4.00 (2.96–5.41)
Sleep duration → Anxiety	5	No downgrade[a]	No downgrade[g]	No downgrade[c]	↓1[h]	Not assessed[i]	**Very Low**[f]	2.14 (1.85–2.46)

Starting level: Low (observational studies).

[a] Most studies achieved moderate-to-high quality on AHRQ/NOS (median score 7/11 and 7/9).

[b] Substantial unexplained heterogeneity: $I^2 = 93.4\%$, wide 95% prediction interval (1.15–13.95).

[c] PECO framework directly matched: exposure (sleep problems), comparator (good sleep), outcome (anxiety symptoms), population (older adults ≥60 years).

[d] Large number of studies ($k = 16$) with narrow pooled CI; total $N = 42{,}977$.

[e] Although Egger's test was significant ($P = 0.028$), trim-and-fill identified only $k_0 = 1$ missing study, and the adjusted OR (3.82, 95% CI: 2.83–5.16) remained highly significant.

[f] Final rating: Low (starting level for observational) − 1 (inconsistency or imprecision) = Very Low.

[g] Moderate heterogeneity: $I^2 = 39.4\%$, acceptable 95% prediction interval (1.46–3.14).

[h] Small number of studies ($k = 5$) with potential sample overlap among CLHLS-derived studies (S131, S151, S130).

[i] Publication bias assessment not feasible with $k < 10$ studies (Egger's/Begg's tests unreliable).

Note: Although the pooled OR of 4.00 exceeds the GRADE threshold for upgrading due to large effect size (OR > 2), we opted not to upgrade because the high heterogeneity (I^2 = 93.4%) and potential residual confounding from unadjusted depressive comorbidity reduce confidence in the precise magnitude of the effect.

TABLE S4. COMPLETE SEARCH STRATEGY FOR ALL DATABASES

Search date: inception to January 2026.

PubMed

Set	Search Terms	Hits
#1	(“sleep duration” OR “sleep quality” OR “PSQI” OR “Pittsburgh Sleep Quality Index” OR “insomnia” OR “sleep disturbance” OR “poor sleep” OR “sleep disorder” OR “sleep problem*”)	101,501
#2	(“anxiety” OR “anxiety symptoms” OR “anxiety disorder*” OR “GAD-7” OR “HADS-A” OR “generalized anxiety” OR “Self-Rating Anxiety Scale” OR “SAS” OR “Beck Anxiety Inventory” OR “anxious”)	433,834
#3	(“older adults” OR “elderly” OR “aged”[MeSH] OR “geriatric” OR “older people” OR “aging” OR “late life”)	4,443,528
#4	#1 AND #2 AND #3	5,002

Web of Science

Set	Search Terms	Hits
#1	TS=(“sleep duration” OR “sleep quality” OR “PSQI” OR “insomnia” OR “sleep disturbance” OR “poor sleep”)	111,951
#2	TS=(“anxiety” OR “anxiety symptoms” OR “anxiety disorder*” OR “GAD-7” OR “GAD” OR “generalized anxiety” OR “HADS-A”)	494,731
#3	TS=(“older adults” OR “elderly” OR “aged” OR “geriatric” OR “older people” OR “late life”)	1,514,393
#4	#1 AND #2 AND #3	3,762

Embase (via Embase.com)

Set	Search Terms	Hits
#1	’sleep duration’/exp	—
#2	’sleep quality’/exp	—
#3	’insomnia’/exp	—
#4	’sleep disorder’/exp	—
#5	’sleep duration’:ti,ab,kw OR ’sleep quality’:ti,ab,kw OR ’sleep time’:ti,ab,kw OR ’psqi’:ti,ab,kw OR ’pittsburgh sleep quality index’:ti,ab,kw OR ’insomnia’:ti,ab,kw OR ’sleep disturbance’:ti,ab,kw OR ’poor sleep’:ti,ab,kw OR ’sleep problem*’:ti,ab,kw	—
#6	#1 OR #2 OR #3 OR #4 OR #5	—
#7	’anxiety’/exp	—
#8	’anxiety disorder’/exp	—
#9	’generalized anxiety disorder’/exp	—
#10	’anxiety’:ti,ab,kw OR ’anxiety symptom*’:ti,ab,kw OR ’anxiety disorder*’:ti,ab,kw OR ’anxious’:ti,ab,kw OR ’gad-7’:ti,ab,kw OR ’hads-a’:ti,ab,kw OR ’self-rating anxiety scale’:ti,ab,kw OR ’sas’:ti,ab,kw OR ’beck anxiety inventory’:ti,ab,kw OR ’generalized anxiety’:ti,ab,kw	—
#11	#7 OR #8 OR #9 OR #10	—
#12	’aged’/exp	—
#13	’very elderly’/exp	—
#14	’geriatric patient’/exp	—
#15	’aging’/exp	—
#16	’older adult*’:ti,ab,kw OR ’elderly’:ti,ab,kw OR ’old people’:ti,ab,kw OR ’aged’:ti,ab,kw OR ’geriatric’:ti,ab,kw OR ’older people’:ti,ab,kw OR ’senior citizen*’:ti,ab,kw OR ’late life’:ti,ab,kw OR ’aging’:ti,ab,kw OR ’ageing’:ti,ab,kw	—
#17	#12 OR #13 OR #14 OR #15 OR #16	—
#18	#6 AND #11 AND #17	—
#19	#18 NOT [medline]/lim	9,585

Cochrane Library

Set	Search Terms	Hits
#1	(“sleep duration” OR “sleep quality” OR “insomnia” OR “PSQI” OR “sleep disturbance” OR “poor sleep”):ti,ab,kw	—
#2	(“anxiety” OR “anxiety symptoms” OR “GAD-7” OR “GAD” OR “generalized anxiety” OR “HADS-A”):ti,ab,kw	—
#3	(“older adults” OR “elderly” OR “aged” OR “geriatric” OR “older people” OR “late life”):ti,ab,kw	—
#4	#1 AND #2 AND #3	16

CNKI (China National Knowledge Infrastructure)

Set	Search Terms	Hits
#1	SU=(sleep duration OR sleep quality OR sleep time OR PSQI OR insomnia OR sleep disorder OR sleep problem OR poor sleep)[a]	—
#2	SU=(anxiety OR anxiety symptoms OR anxiety disorder OR GAD OR generalized anxiety)[a]	—
#3	SU=(older adults OR elderly OR aged OR aging OR geriatric)[a]	—
#4	#1 AND #2 AND #3	658

[a] Searches were conducted using the equivalent Chinese terms in CNKI.

Total records identified across all databases: 19,023. After removing 17,171 duplicates, 1,852 unique records were screened.

TABLE S5. SUMMARY OF EXCLUDED STUDIES AFTER FULL-TEXT ASSESSMENT

A total of 219 full-text articles were excluded during the eligibility assessment. The reasons for exclusion are summarized below. The complete list of excluded studies with individual citations is available from the corresponding author upon reasonable request.

Exclusion Reason	n	%	Description
No extractable OR + 95% CI	53	24.2	Only reported correlation coefficients (r), regression coefficients (β), mean comparisons, or structural equation models without direct OR
Non-observational study design	46	21.0	Interventional studies (RCT), reviews, meta-analyses, systematic reviews, conference abstracts
Non-sleep exposure	46	21.0	Primary exposure was diet, pain, physical activity, social factors, or other non-sleep variables
Anxiety not primary outcome	38	17.4	Anxiety served as covariate, mediator, or exposure rather than outcome; or reverse direction (anxiety $\rightarrow$ sleep)
Disease-specific population	18	8.2	Studies restricted to patients with cancer, epilepsy, COPD, post-surgical, or other specific clinical populations
Overlapping sample	1	0.5	Yang X (2017): same sample as Tang R (2019) from PLA General Hospital; retained Tang R (2019)
Other	17	7.8	Non-elderly population (mean age $<$60 years), duplicate publication, non-validated anxiety measure
**Total**	**219**	**100**	

Note: One study (Yang X, 2017) was excluded due to overlapping sample with an included study (Tang R, 2019); both originated from the PLA General Hospital retired male cohort ($N = 1{,}132$). The study with more comprehensive data was retained.

SUPPLEMENTARY FIGURES

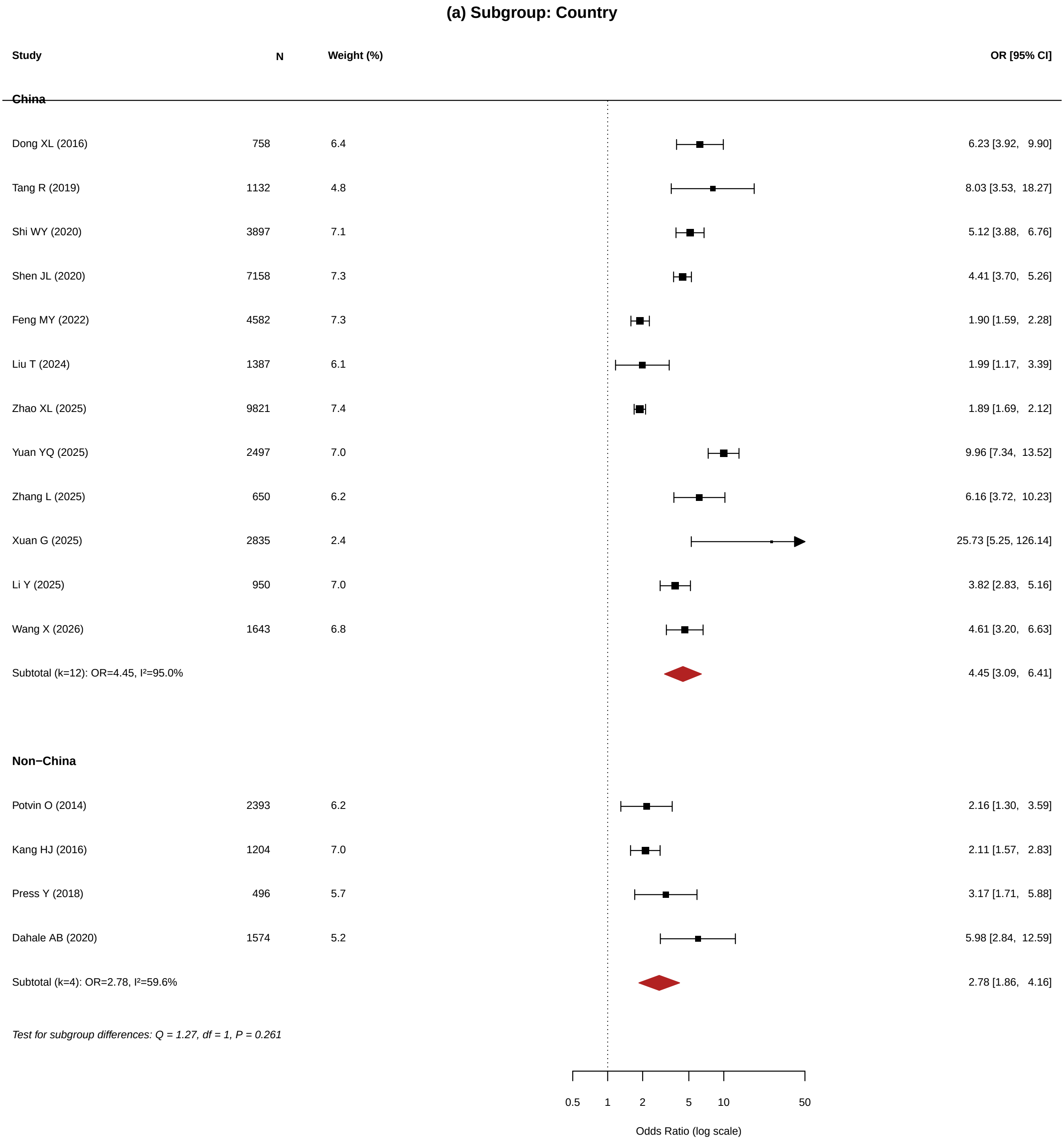

Figure 1. Forest plot of subgroup analyses for the association between sleep quality and anxiety symptoms in older adults. Subgroups were defined by geographic region (China vs. Non-China), study design (cross-sectional vs. cohort), quality rating (high vs. moderate), anxiety scale (GAD vs. SAS vs. Other), and exposure tool (PSQI vs. Insomnia-specific vs. Single-item). $P_{\text{interaction}}$ values from Cochran's Q test for subgroup differences are shown for each dimension.

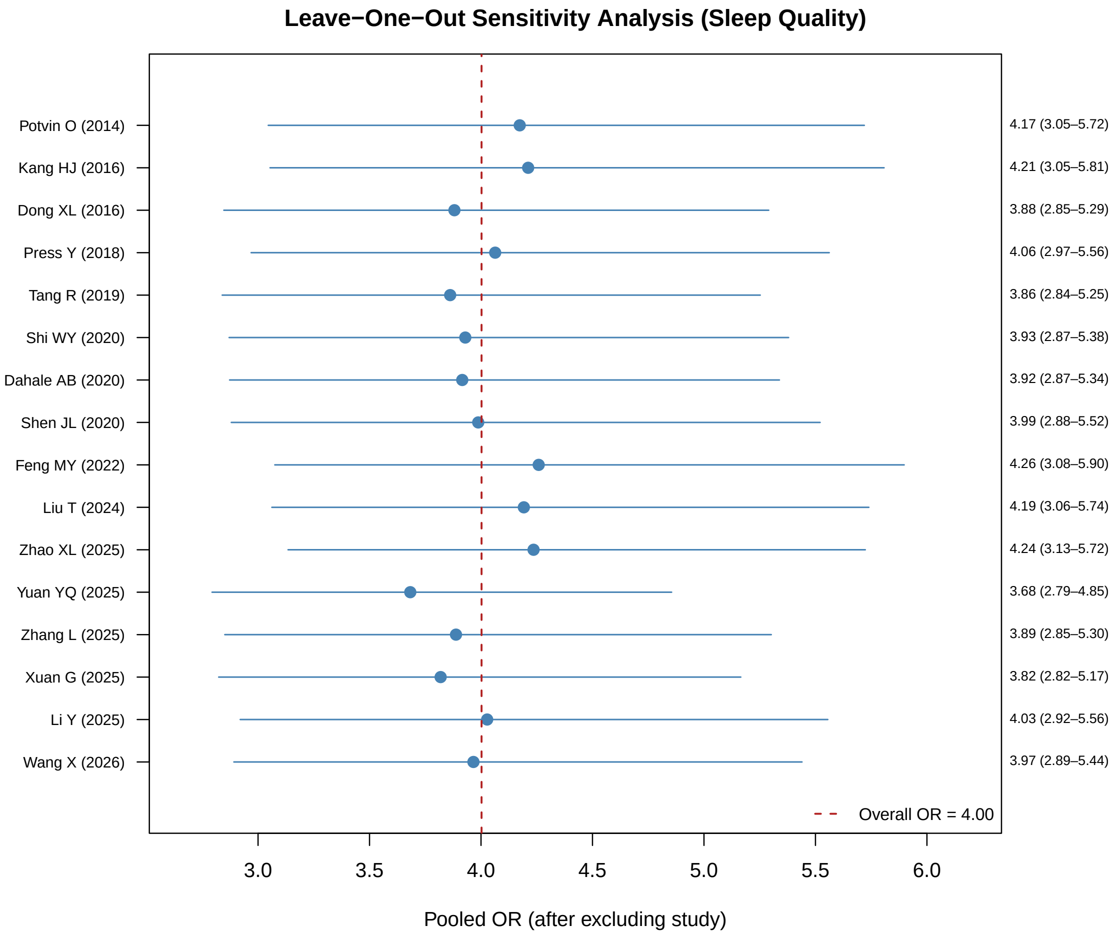

Figure 2. Leave-one-out sensitivity analysis for the sleep quality–anxiety meta-analysis ($k = 16$). Each row shows the pooled OR (95% CI) after removing the named study. The pooled OR ranged from 3.68 (excluding S83 Yuan YQ 2025) to 4.26 (excluding S148 Feng MY 2022), indicating that no single study disproportionately influenced the overall estimate.

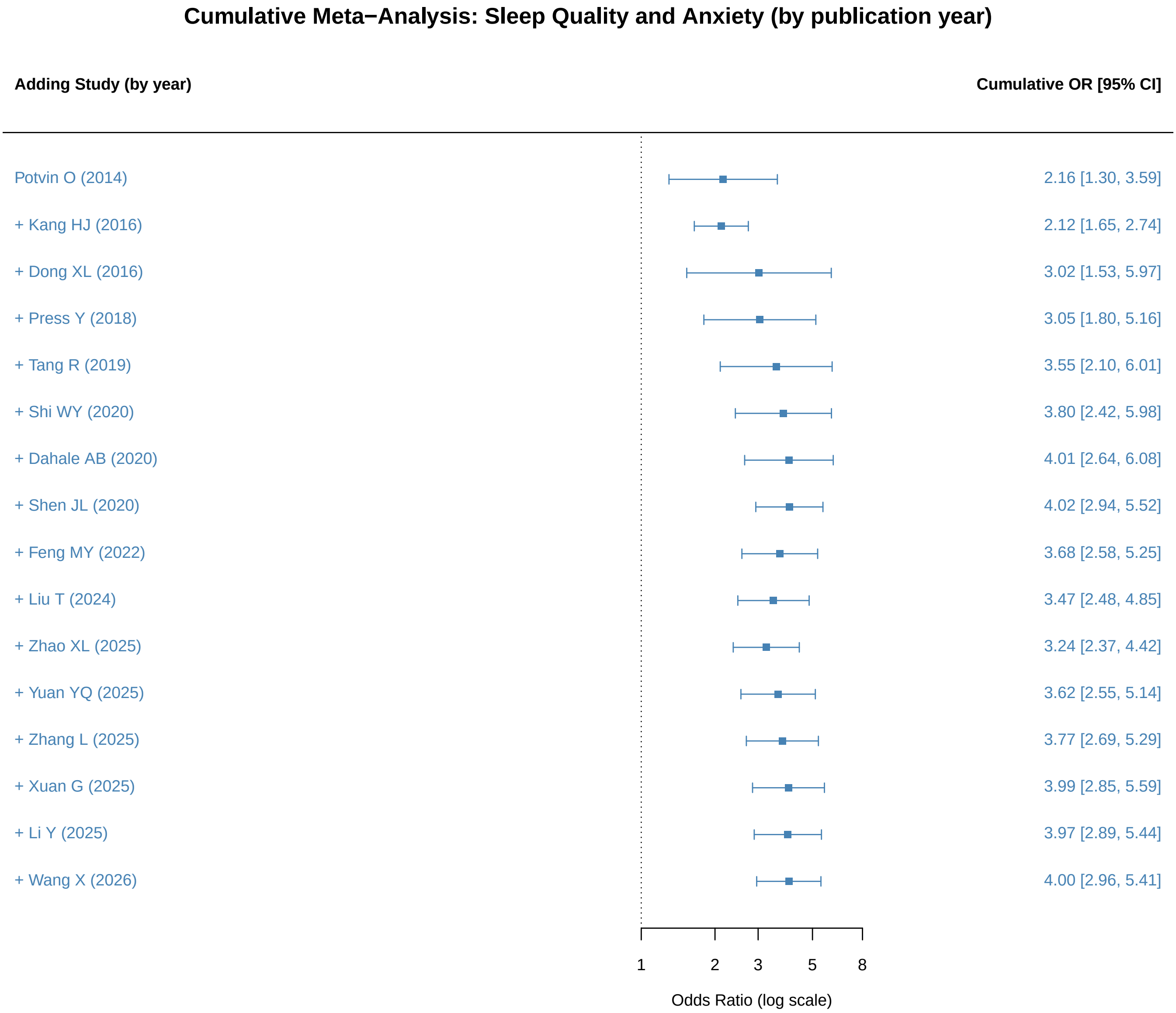

Figure 3. Cumulative meta-analysis for the sleep quality–anxiety association, ordered by publication year (2014–2026). The pooled OR stabilized within the approximate range of 3.0–4.0 after the accumulation of $\geq$7 studies (from 2020 onward), demonstrating temporal robustness of the overall effect estimate.

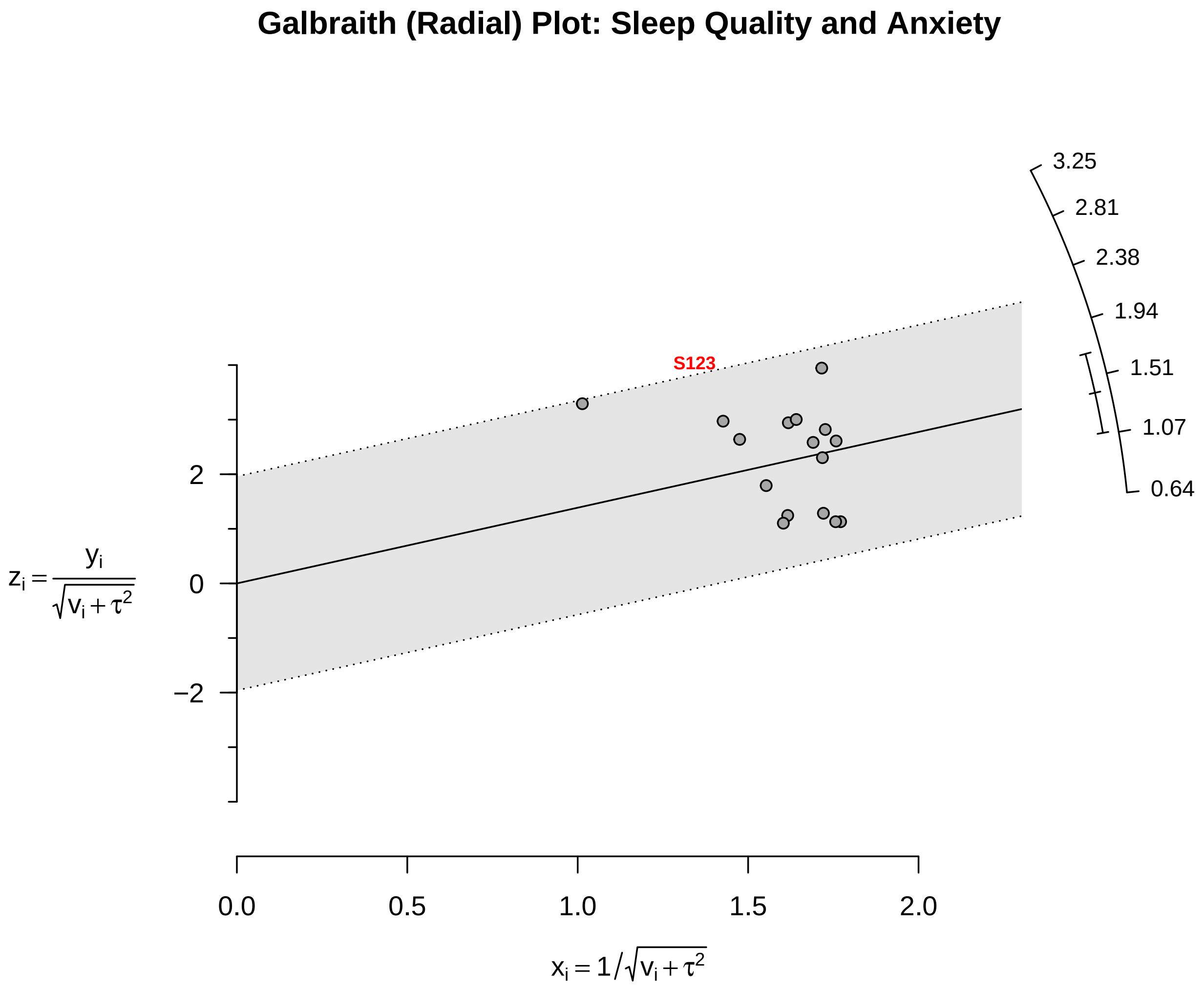

Figure 4. Galbraith (radial) plot for the sleep quality–anxiety meta-analysis ($k = 16$). Studies falling outside the ± 2 confidence band are potential sources of heterogeneity. The plot visually identifies S123 (Xuan G 2025) and S83 (Yuan YQ 2025) as the primary outliers contributing to the high I^2 (93.4%).
